# Canine vaccination in Germany: A survey of owner attitudes and compliance

**DOI:** 10.1371/journal.pone.0238371

**Published:** 2020-08-27

**Authors:** Simone Eschle, Katrin Hartmann, Anna Rieger, Sebastian Fischer, André Klima, Michèle Bergmann

**Affiliations:** 1 Clinic of Small Animal Medicine, Centre for Clinical Veterinary Medicine, Ludwig-Maximilians-University Munich, Munich, Bavaria, Germany; 2 Department of Statistics, Ludwig-Maximilians-University Munich, Munich, Bavaria, Germany; University of Lincoln, UNITED KINGDOM

## Abstract

**Background:**

Vaccination is the most important preventive measure for protection against infectious diseases in humans and companion animals. Nevertheless, scepticism about the safety and importance of vaccines is increasing in human and in veterinary medicine. Although owner attitudes towards vaccination have been investigated in cats, there are no similar studies in dogs. The goals of this study were therefore to investigate the vaccination status of dogs in Germany, to determine owner compliance with vaccination and to identify factors that play a role in owners’ decisions to have their dogs vaccinated.

**Methods:**

Data were collected from August 2018 to February 2019 using an online survey targeting dog owners in Germany. A total of 3,881 questionnaires were evaluated, and factors associated with the vaccination status of dogs were determined by a linear logistic regression model using Akaike information criterion. Cohen's kappa statistic was used to evaluate agreement between questionnaire and 340 vaccination passports submitted voluntarily by owners.

**Results:**

A total of 46.8% (n = 1,818/3,881) of dogs were vaccinated with core vaccines according to current guidelines with the lowest vaccination rate for leptospirosis (50.1%; n = 1,941/3,874). Dog’s age (16 weeks to 15 months) (odds ratio (OR): 3.08; 95% CI: 2.05–4.68), type (working dog) (OR: 2.06; 95% CI: 1.22–3.53) and travelling abroad within previous 36 months (OR: 1.82; 95% CI: 1.12–2.96) had the strongest ‘positive’ association with the vaccination status. Recommendation from a veterinarian not to vaccinate against leptospirosis had the strongest ‘negative’ association (OR: 0.08; 95% CI: 0.04–0.18).

**Conclusion:**

The study revealed a need for improvement in vaccination compliance because of inadequate vaccination coverage, especially for leptospirosis, in dogs. Factors influencing owner compliance were numerous. Vaccination recommendations made by the veterinarian had a strong association with the vaccination status and should be used to increase canine vaccination rates.

## Introduction

Vaccination is considered one of the most important measures of preventing and controlling human and animal infectious diseases [1, 2]. A distinction is made between core and non-core vaccines in dogs. Core vaccines protect against infectious diseases that are common and have a severe outcome or zoonotic potential. They should be administered to all dogs. Non-core vaccines provide protection against specific infectious diseases and are given to dogs based on their risk of infection, which is influenced by factors, such as geographical location and lifestyle [3, 4].

According to the current World Small Animal Veterinary Association (WSAVA) vaccination guidelines, vaccines against canine distemper virus (CDV), canine adenovirus (CAV-1 and CAV-2) and canine parvovirus (CPV) are considered core vaccines in dogs. Rabies is a core vaccine in countries where it is endemic or required by law [4]. Since 2009, German guidelines have included vaccination against *Leptospira* spp. as a core component due to increase in prevalence in Germany and neighbouring countries and the zoonotic potential of the disease [5]. Due to zoonotic nature of leptospirosis [6], it is crucial to prevent humans from getting the infection through controlling the disease in dogs. Given that cases of hepatitis contagiosa canis (caused by CAV-1) only sporadically occur in Western Europe and that CAV-2 causes mild infection in dogs suffering from canine infectious respiratory disease complex (CIRDC), vaccination against CAV is a non-core vaccine.

To avoid epizootics, a population must reach a certain threshold of immunity, referred to as 'herd immunity', which describes how the ratio between the number of individuals immune to a particular infectious disease and the number of non-immune individuals influences the rate at which the infectious disease spreads. The equation to describe ‘herd immunity’ is (R0-1)/R0 [7]; the ‘basic reproduction number’ (R0) is defined as the average number of secondary cases caused by a single case within a susceptible population [8]. For example, the R0 for rabies in dogs in Machakos District, Kenya, represented 2.44 [9] and thus 59% of individuals within the dog population needed to be protected to avoid an epizootic. To the authors’ knowledge, there are no R0 values published for pathogens against which dogs are vaccinated, with the exception of rabies. Therefore, a vaccination coverage of ≥75% is generally recommended for all core vaccines [4]. Owner compliance with vaccination recommendations is crucial to achieve this level of vaccination coverage.

Diseases such as canine distemper and parvovirosis are still present in Northern and Western Europe [10, 11], although they are observed less frequently, but the exact number of clinical cases is unknown. Nevertheless, repeated outbreaks occur, mainly due to importation of dogs from Southern or Eastern European countries [12–14]. Therefore all dogs should have an up-to-date vaccination status. In human medicine, people are increasingly concerned about vaccine-associated adverse effects (VAAEs) and are unsure about using vaccines for themselves or for their children [15]. Similarly, many animal owners are hesitant about vaccinating their pets, they doubt the necessity of vaccinations [16] and are concerned about VAAEs [17, 18]. Several factors including waiting times at veterinary clinics have been shown to negatively affect owner compliance with cat vaccination in Germany [18]. The results of that study can be used to improve vaccination coverage in cats, and a similar study would be useful for the same purpose in dogs.

Currently, dog population in Germany is estimated to be 9.4 million and 20% of veterinary visits consist of preventive health care, including vaccinations [19]. However, there are no studies on dogs’ vaccination rates and owners’ attitudes to vaccination in Germany so far. Studies on compliance of dog owners across Europe are also scarce. In a 2019 survey (Animal Wellbeing Report) conducted in United Kingdom (UK), 78% of dogs received regular vaccination, but details on vaccination intervals and the vaccines used were not provided. Owners who were non-compliant stated that vaccination would be unnecessary and too expensive [20]. In a study done in Uganda on dog owners’ attitudes towards rabies vaccination, it was determined that 55.6% of owned dogs received rabies vaccination and poverty had a negative impact on vaccination rate [21]. However, the socio-demographic differences between Uganda and Germany likely indicate that the factors associated with owner compliance differ, and thus it is necessary to conduct a separate study focussing on dog owners in a European country.

The aim of the present study was to determine owner compliance with vaccination guidelines recommended for dogs. The status of dogs with regard to core vaccines and the factors associated with owner compliance were determined.

## Methods

### Ethics and data protection

The study was approved by the ethical committee of the Centre for Veterinary Clinical Medicine, LMU Munich, Germany (reference number 140-25-07-2018). Data collection was carried out in accordance with the European General Data Protection Regulation (GDPR), and owner consent was obtained by an opt-in procedure. Email addresses that were provided voluntarily were deleted, and the data anonymised immediately after assigning the vaccination passports to the corresponding questionnaires and completing the evaluation of the vaccination passports.

### Data collection

A web-based questionnaire was developed based on the one designed for cat owners [18]. The questionnaire consisted of 3 parts: (1) general information about dogs including history of vaccinations and attitudes of owners towards canine vaccination, (2) the occurrence of canine infectious diseases in the owners’ household and previous VAAEs, (3) the sociodemographic background and vaccination status of owners, their children and attitudes towards human vaccination. The questionnaire was written in German (S1 Questionnaire) and the survey was translated into English (S2 Questionnaire) for this publication. The survey was published online using EVASys V7.1 (Electric Paper Evaluationssysteme GmbH, Lüneburg) from August 2018 to February 2019. The questionnaire targeted dog owners in Germany. A link to the survey was published on the homepage of the Clinic of Small Animal Medicine, LMU Munich, across various special interest platforms, such as dog forums and online trade journals, and was also distributed via social media, e. g., Facebook. Using the snowball effect, participants were able to share the link and send it to others. Owners <16 years of age, owners who did not indicate their age, owners of dogs <8 weeks of age, veterinarians and owners living outside Germany were excluded from further evaluation. Demographic details of the participants recruited are summarised in S1 Table.

The results are not necessarily generally representative of the dog population or of dog owners in Germany because of the online nature of the survey.

### Vaccination passports

After completion of the survey, owners were asked to voluntarily submit a copy of the vaccination passport of their dog. Passports were compared with the survey answers to verify the statements of the owners relating to the vaccination status of their dog. As compensation, owners who sent passport copies received a free consultation on the vaccination history of their dog and recommendations for an optimal vaccination schedule according to current guidelines for dogs.

### Vaccination status

For the purpose of this study, dogs were classified as having an ‘up-to-date vaccination status’ when the owner reported valid vaccination of all core vaccines according to the national vaccination guidelines at the time of the survey. This included vaccination against CDV, CPV and rabies virus (RV) within the previous 3 years and against *Leptospira* spp. within the previous 12 months.

### Data analysis

To determine potential factors with an effect on vaccination status, the dependent variable ‘up-to-date vaccination status’ was defined. Reference categories were determined according to their content and represented the response options that were expected to be chosen by most dog owners. For the model, a binary generalised linear model (GLM) with logit link was used. A stepwise backwards model selection was done based on the Akaike information criterion (AIC) [22], using R Version 3.4.4. Since model selection leads to the final model formula, a post selection inference is caused. This affects the validity of p-values, which are therefore not presented. In order to provide an indication of the estimation uncertainty, the confidence intervals are presented, but for the same reason they should be interpreted with caution. For the presentation of the results, factors were ranked according to the odds ratio (OR), whereby OR >1 expressed an increased expected probability and OR <1 expressed a decreased expected probability of 'up-to-date vaccination status' of the dogs in relation to their respective reference categories. Based on this, the OR were classified as ‘positive’ and ‘negative’ influencing factors with regard to their content-related reference categories. To assess agreement between information relating to the survey and the vaccination passports, Cohen´s Kappa statistic was calculated.

## Results

### Survey response

A total of 4,202 questionnaires were completed and those from participants who met exclusion criteria were left out: 3,881 questionnaires were evaluated. Table 1 shows the variables investigated in the logistic regression and selected by the model. The variables that were not selected and thus eliminated by the model are listed in S2–S5 Tables. Table 2 shows the vaccination history of the dogs and owner attitudes towards vaccination.

**Table 1:** Variables investigated and selected by the logistic regression model (n = 3,881).

Question	Response option		Frequency of responses	Percentage of responses	Final model results (based on Akaike information criterion)	
					OR	95% CI
Number of dogs owned by respondent	1		2,005/3,872	51.8	1.00	Ref. value
	2		1,108/3,872	28.6	1.12	0.89–1.41
	3		392/3,872	10.1	0.83	0.58–1.19
	≥4		367/3,872	9.5	0.69	0.46–1.04
Age of the dog	8 to 16 weeks		37/3,881	1.0	1.00	0.36–2.86
	16 weeks to 15 months		294/3,881	7.6	3.08	2.05–4.68
	15 months to 5 years		1,467/3,881	37.8	1.00	Ref. value
	5 to 10 years		1,430/3,881	36.8	0.56	0.44–0.70
	≥10 years		633/3,881	16.3	0.39	0.28–0.53
	unknown		20/3,881	0.5	0.47	0.09–2.37
Frequency of swimming	Never		909/3,860	23.5	0.73	0.56–0.95
	Rare, ≤ once a week		1,394/3,860	36.1	0.86	0.69–1.07
	Often, > once a week		1,557/3,860	40.3	1.00	Ref. value
Type of dog	Family dog	Yes	3,693/3,881	95.2	1.00	Ref. value
		No	188/3,881	4.8	1.58	0.95–2.63
	Working dog	Yes	137/3,881	3.5	2.06	1.22–3.53
		No	3,744/3,881	96.5	1.00	Ref. value
Boarding kennel or dog sitter (in previous 24 months)	Yes		601/3,881	15.5	0.66	0.47–0.92
	No		3,187/3,881	82.1	1.00	Ref. value
	Unknown		93/3,881	2.4	1.71	0.76–3.93
Last stay abroad	Within the previous 12 months		1,419/3,870	36.7	1.25	1.01–1.55
	Within the previous 24 months		311/3,870	8.0	1.33	0.92–1.91
	Within the previous 36 months		172/3,870	4.4	1.82	1.12–2.96
	>36 months ago		1,968/3,870	50.9	1.00	Ref. value
Planned outing in the next 36 months	Boarding kennel or dog sitter	Yes	392/3,702	10.6	1.79	1.18–2.74
		No	3,008/3,702	81.3	1.00	Ref. value
		Unknown	302/3,702	8.2	1.11	0.77–1.60
	Dog show	Yes	415/3,689	11.2	1.54	1.07–2.24
		No	3,032/3,689	82.2	1.00	Ref. value
		Unknown	242/3,689	6.6	1.21	0.82–1.79
	Dog training	Yes	1,179/3,759	31.4	1.42	1.13–1.80
		No	2,131/3,759	56.7	1.00	Ref. value
		Unknown	449/3,759	11.9	1.23	0.90–1.69
Source of vaccination information	Veterinarian	Very helpful	1,390/3,859	36.0	1.00	Ref. value
		Helpful	1,240/3,859	32.1	0.66	0.52–0.85
		Not helpful	1,002/3,859	26.0	0.39	0.29–0.53
		Source not used	227/3,859	5.9	0.39	0.24–0.63
	Homeopathic practitioner	Very helpful	336/3,746	9.0	0.37	0.24–0.57
		Helpful	431/3,746	11.5	0.39	0.27–0.56
		Not helpful	314/3,746	8.4	0.64	0.44–0.94
		Source not used	2,665/3,746	71.1	1.00	Ref. value
	Books and magazines	Very helpful	376/3,753	10.0	0.65	0.43–0.98
		Helpful	1,043/3,753	27.8	0.84	0.66–1.06
		Not helpful	389/3,753	10.4	1.28	0.90–1.83
		Source not used	1,945/3,753	51.8	1.00	Ref. value
Owner’s knowledge about vaccination	Excellent		1,574/3,864	40.7	1.00	Ref. value
	Average		1,682/3,864	43.5	1.00	0.79–1.28
	Somewhat poor		520/3,864	13.5	0.85	0.61–1.18
	Poor		88/3,864	2.3	0.24	0.10–0.53
Recommendations of veterinarians on revaccination	*Leptospira* spp.	Yearly	2,191/3,861	56.7	1.00	Ref. value
		Every 2 years	188/3,861	4.9	0.61	0.38–0.98
		Every 3 years or less frequently	152/3,861	3.9	0.27	0.15–0.47
		Only when needed	146/3,861	3.8	0.29	0.14–0.62
		Never	221/3,861	5.7	0.08	0.04–0.18
		Unknown	963/3,861	24.9	0.21	0.16–0.28
	Rabies virus	Yearly	737/3,870	19.0	0.57	0.43–0.74
		Every 2 years	431/3,870	11.1	1.11	0.80–1.53
		Every 3 years	2,146/3,870	55.5	1.00	Ref. value
		Less frequently than every 3 years	41/3,870	1.1	0.54	0.11–1.96
		Only when needed (e. g., after antibody measurement)	105/3,870	2.7	1.09	0.47–2.48
		Never	103/3,870	2.7	0.71	0.22–2.09
		Unknown	307/3,870	7.9	1.75	1.14–2.72
	CDV and CPV	Yearly	1,721/3,871	44.5	1.78	1.29–2.45
		Every 2 years	338/3,871	8.7	1.81	1.15–2.86
		Every 3 years	619/3,871	16.0	1.00	Ref. value
		Less frequently than every 3 years	54/3,871	1.4	0.21	0.04–0.72
		Only when needed (e. g., after antibody measurement)	192/3,871	5.0	0.59	0.28–1.19
		Never	144/3,871	3.7	1.24	0.48–3.13
		Unknown	803/3,871	20.7	0.90	0.59–1.35
Factors influencing owner’s decision to have the dog vaccinated	VAAEs	Unimportant	135/3,871	3.5	0.76	0.44–1.32
		Somewhat unimportant	223/3,871	5.8	1.36	0.87–2.15
		Somewhat important	642/3,871	16.6	1.50	1.10–2.03
		Important	727/3,871	18.8	0.93	0.71–1.22
		Very important	2,144/3,871	55.4	1.00	Ref. value
	Veterinary consultation	Unimportant	703/3,836	18.3	0.52	0.36–0.75
		Somewhat unimportant	375/3,836	9.8	0.70	0.48–1.02
		Somewhat important	905/3,836	23.6	0.86	0.65–1.13
		Important	725/3,836	18.9	1.08	0.81–1.44
		Very important	1,128/3,836	29.4	1.00	Ref. value
	Severity of the diseases	Unimportant	341/3,842	8.9	1.50	0.96–2.34
		Somewhat unimportant	118/3,842	3.1	0.90	0.48–1.65
		Somewhat important	426/3,842	11.1	0.73	0.52–1.03
		Important	721/3,842	18.8	0.74	0.57–0.96
		Very important	2,236/3,842	58.2	1.00	Ref. value
	Age of the dog	Unimportant	592/3,857	15.3	1.23	0.89–1.70
		Somewhat unimportant	320/3,857	8.3	1.46	0.99–2.15
		Somewhat important	849/3,857	22.0	1.62	1.23–2.13
		Important	720/3,857	18.7	1.12	0.84–1.50
		Very important	1,376/3,857	35.7	1.00	Ref. value
Potential deterrents from making a veterinary appointment	Others	Yes	248/3,881	6.4	0.71	0.46–1.09
		No	3,633/3,881	93.6	1.00	Ref. value
Discouraged from vaccinations by VAAEs	Yes		826/3,860	21.4	0.35	0.26–0.49
	No		780/3,860	20.2	0.97	0.76–1.23
	No VAAEs in the past		2,254/3,860	58.4	1.00	Ref. value
Gender of the owner	Female		3,606/3,847	93.7	1.00	Ref. value
	Male		241/3,847	6.3	0.74	0.50–1.11
Attitude of the respondent towards vaccinations	Very important to indispensable		1,279/3,872	33.0	1.00	Ref. value
	Generally positive but decision made after considering advantages and disadvantages		1,921/3,872	49.6	0.59	0.46–0.74
	Somewhat sceptical		555/3,872	14.3	0.38	0.26–0.57
	Opposed to all types of vaccinations		117/3,872	3.0	0.40	0.15–0.95
Respondent was vaccinated in the previous 10 years	Tetanus	Yes	3,096/3,874	79.9	1.58	1.14–2.19
		No	696/3,874	18.0	1.00	Ref. value
		Unknown	82/3,874	2.1	0.80	0.38–1.65
	Diphtheria	Yes	1,658/3,866	42.9	0.89	0.70–1.12
		No	1,815/3,866	46.9	1.00	Ref. value
		Unknown	393/3,866	10.2	0.64	0.45–0.91

VAAEs = vaccine-associated adverse effects; OR = odds ratio; CI = confidence interval; Ref. value = reference value; CPV = canine parvovirus; CDV = canine distemper virus.

Of all factors included in the statistical analysis, 27 factors were selected by the model; p-values were omitted because of post-selection inference (Table 1).

**Table 2:** Vaccination history of dogs and owner attitudes towards vaccination (n = 3,881).

Variable	Response option	Frequency of responses	Percentage of responses
Dog received vaccinations in the past	Yes	3,811/3,851	99.0
	No	40/3,851	1.0
‘Up-to-date vaccination status’	Yes	1,818/3,881	46.8
	No	2,063/3,881	53.2
Last leptospirosis vaccination	Within the previous year	1,941/3,874	50.1
	More than 1 year to 3 years ago	744/3,874	19.2
	More than 3 years ago	537/3,874	13.9
	Never	303/3,874	7.8
	Unknown	349/3,874	9.0
Last rabies vaccination	Within the previous year	1,855/3,872	47.9
	More than 1 year to 3 years ago	1,488/3,872	38.4
	More than 3 years ago	377/3,872	9.7
	Never	109/3,872	2.8
	Unknown	43/3,872	1.1
Last distemper and parvovirosis vaccination	Within the previous year	1,888/3,863	48.9
	More than 1 year to 3 years ago	1,027/3,863	26.6
	More than 3 years ago	647/3,863	16.7
	Never	89/3,863	2.3
	Unknown	212/3,863	5.5
Type of VAAEs*	Lethargy	1,128/1,613	69.9
	Anorexia	517/1,613	32.1
	Reactions at injection site	990/1,613	61.4
	Fever	490/1,613	30.4
	Vomiting	378/1,613	23.4
	Diarrhoea	574/1,613	35.6
	Lameness	207/1,613	12.8
	Anaphylactic reaction (within 24 hours)	271/1,613	16.8
	Immune mediated disease	117/1,613	7.3
	Other	260/1,613	16.1
Assessment of VAAEs in the past*	Not significant and rare	414/1,585	26.1
	Not significant and often	297/1,585	18.7
	Significant and rare	473/1,585	29.8
	Significant and often	401/1,585	25.3
Assessment of the importance of vaccination: leptospirosis	Very important	1,397/3,844	36.3
	Important	1,075/3,844	28.0
	Somewhat important	522/3,844	13.6
	Unimportant	442/3,844	11.5
	Unknown	408/3,844	10.6
Assessment of the importance of vaccination: canine distemper	Very important	2,231/3,855	57.9
	Important	1,069/3,855	27.7
	Somewhat important	255/3,855	6.6
	Unimportant	195/3,855	5.1
	Unknown	105/3,855	2.7
Assessment of the importance of vaccination: parvovirosis	Very important	2,244/3,844	58.4
	Important	996/3,844	25.9
	Somewhat important	236/3,844	6.1
	Unimportant	194/3,844	5.0
	Unknown	174/3,844	4.5
Assessment of the importance of vaccination: rabies	Very important	2,232/3,853	57.9
	Important	927/3,853	24.1
	Somewhat important	414/3,853	10.7
	Unimportant	254/3,853	6.6
	Unknown	26/3,853	0.7
Reasons against vaccination*	Vaccinations are needless and unnecessary	53/117	45.3
	Vaccinations are harmful to health; they weaken the immune system	99/117	84.6
	Vaccinations can trigger other illnesses	96/117	82.1
	Vaccinations benefit only doctors and the pharmaceutical industry	79/117	67.5
	Other reasons	12/117	10.3

‘Up-to-date vaccination status’: the dog received the last rabies, distemper, and parvovirosis vaccination within the previous 3 years, and the last leptospirosis vaccination within the previous year.

*Filtered Question: This question was only visible when the previous question was answered accordingly.

Some factors were evaluated descriptively and not included in the statistical model (Table 2).

### Vaccination status of the dogs

Of all respondents, 46.8% (n = 1,818/3,881) considered their dogs ‘up-to-date on vaccinations’, whilst 1.0% (n = 40/3,851) owned dogs that never received vaccination. Within the previous 3 years, 86.3% and 75.5% of the dogs received RV (n = 3,343/3,872) and CDV/CPV (n = 2,915/3,863) vaccinations, respectively. Around 50.1% (n = 1,941/3,874) of the dogs were vaccinated against *Leptospira* spp. within the previous 12 months (Table 2).

### Recommendations of veterinarians on vaccination

As reported by owners, 16.0% (n = 619/3,871) of veterinarians recommended triennial vaccinations against CDV/CPV, whilst 53.2% (n = 2,059/3,871) recommended annual and biennial vaccinations. Nearly 55.5% (n = 2,146/3,870) of veterinarians recommended triennial RV vaccinations, whilst 56.7% (n = 2,191/3,861) advised annual vaccination against *Leptospira* spp. (Table 1).

### Vaccination records

Of all owners, 8.8% (n = 340/3,881) submitted the vaccination passports of their dogs. The descriptive evaluation of the passports and the survey results were similar (Table 3). Nearly 67.9% and 58.2% of the passports and the questionnaires showed that the dogs had an ‘up-to-date vaccination status’, respectively. The vaccination status reported on the questionnaires and vaccination passports did not match in 19.1% (n = 65/340) of the dogs; the kappa coefficient of 0.594 with a p-value of <0.001 indicated lack of agreement between the two assessments. A total of 4.7% (n = 16/340) of owners overestimated the vaccination status of their dog, and 14.4% (n = 49/340) of owners underestimated the status. In 80.9% (n = 275/340) of dogs, the vaccination status obtained from the questionnaire was consistent with the vaccination status of the vaccination passport. Around 53.5% (n = 182/340) of owners correctly stated that the dog had an ‘up-to-date vaccination status’ whilst 27.4% (n = 93/340) correctly stated that the dog did not have an ‘up-to-date vaccination status’.

**Table 3:** Comparison of data from vaccination passports and survey responses (n = 340).

	Owners’ responses (n = 340)		Data from canine vaccination passports (n = 340)	
	Frequency	Percent	Frequency	Percent
**Dog received vaccinations in the past**				
Yes	337	99.1	340	100.0
No	3	0.9	0	0.0
**Last leptospirosis vaccination**				
Within the previous year	207	60.9	245	72.1
More than 1 year ago	84	24.7	91	26.8
Never	7	2.1	4	1.2
Unknown	41	12.1	-	-
Not answered	1	0.3	-	-
**Last rabies vaccination**				
Within the previous 3 years	311	91.5	314	92.4
More than 3 years ago	13	3.8	21	6.2
Never	5	1.5	5	1.5
Unknown	8	2.4	-	-
Not answered	3	0.9	-	-
**Last distemper and parvovirosis vaccination**				
Within the previous 3 years	284	83.5	290	85.3
More than 3 years ago	24	7.1	46	13.5
Never	1	0.3	4	1.2
Unknown	29	8.5	-	-
Not answered	2	0.6	-	-
**‘Up-to-date vaccination status’**				
Yes	198	58.2	231	67.9
No	142	41.7	109	32.1

‘Up-to-date vaccination status’: the dog received the last rabies, distemper, and parvovirosis vaccinations within the previous 3 years, and the last leptospirosis vaccination within the previous year.

The comparison of the survey results with the submitted vaccination certificates shows almost complete agreement for the current rabies, distemper and parvovirus vaccination status, whilst more dogs had a current leptospirosis vaccination status than stated in the questionnaires (Table 3).

### Factors associated with status of canine vaccination in Germany

Twenty-seven factors were selected by the statistical model and thus associated with ‘up-to-date vaccination status’ reported by the owner. Dog’s age (16 weeks to 15 months) (OR: 3.08; 95% CI: 2.05–4.68), type (working dogs; OR: 2.06; 95% CI: 1.22–3.53) and travelling abroad within the previous 36 months (OR: 1.82; 95% CI: 1.12–2.96) were the factors having strongest ‘positive’ association with an ‘up-to-date vaccination status’ in relation to their respective reference categories (Table 4).

**Table 4:** The 10 factors with the strongest ‘positive’ association with an 'up-to-date vaccination status' in relation to the respective reference categories (n = 3,881).

Factors	Reference category	Dogs per category		Final model results (based on AIC)	
		Frequency	Percent	OR	95% CI
Age of the dog (16 weeks to 15 months)	Age of the dog (15 months to 5 years)	294/3,881	7.6	3.08	2.05–4.68
Type of dog (working dog)	Type of dog (not a working dog)	137/3,881	3.5	2.06	1.22–3.53
Travelling abroad within the previous 36 months	Travelling abroad >36 months ago	172/3,870	4.4	1.82	1.12–2.96
CDV and CPV revaccination recommended by veterinarian (every 2 years)	CDV and CPV revaccination recommended by veterinarian (every 3 years)	338/3,871	8.7	1.81	1.15–2.86
Stay in a boarding kennel within the next 36 months (yes)	Stay in a boarding kennel within the next 36 months (no)	392/3,702	10.6	1.79	1.18–2.74
CDV and CPV revaccination recommended by veterinarian (every year)	CDV and CPV revaccination recommended by veterinarian (every 3 years)	1,721/3,871	44.5	1.78	1.29–2.45
RV revaccination recommended by veterinarian (unknown)	RV revaccination recommendation by veterinarian (every 3 years)	307/3,870	7.9	1.75	1.14–2.72
Stay in a boarding kennel in the previous 24 months (unknown)	Stay in a boarding kennel in the previous 24 months (no)	93/3,881	2.4	1.71	0.76–3.93
Age of dog (somewhat important) as a factor influencing vaccination decision by the owner	Age of dog (very important) as a factor influencing vaccination decision by the owner	849/3,857	22.0	1.62	1.23–2.13
Respondent was vaccinated against tetanus in the previous 10 years	Respondent was not vaccinated against tetanus in the previous 10 years	3,096/3,874	79.9	1.58	1.14–2.19

OR = odds ratio; CI = confidence interval; AIC = Akaike information criterion; CPV = canine parvovirus; CDV = canine distemper virus; RV = rabies virus.

Factors associated with the vaccination status of the dogs resulted from a logistic regression after stepwise model selection based on the Akaike Information Criterion (AIC); p-values were omitted because of post-selection inference. The definition of the reference categories was made on a case-by-case basis; the option that was expected to reflect the response of most owners was chosen. Compared with this reference category, the expected probability of a variable in a dog with an ‘up-to-date vaccination status’ increases with increasing odds ratio (OR); these variables were therefore referred to as ‘positive’ influencing factors (Table 4).

Regarding veterinary recommendations on vaccinating dogs against infectious diseases, advice not to vaccinate against *Leptospira* spp. (OR: 0.08; 95% CI: 0.04–0.18), to vaccinate against CDV/CPV less frequently than every 3 years (OR: 0.21; 95% CI: 0.04–0.72) and owner’s ignorance of regular revaccination against leptospirosis (OR: 0.21; 95% CI: 0.16–0.28) had the strongest ‘negative’ association with ‘up-to-date vaccination status’ of a dog in relation to their respective reference categories (Table 5).

**Table 5:** The 10 factors with the strongest ‘negative’ association with an ' up-to-date vaccination status' in relation to the respective reference categories (n = 3,881).

Factors	Reference category	Dogs per category		Final model results (based on AIC)	
		Frequency	Percent	OR	95% CI
*Leptospira* spp. revaccination recommended by veterinarian (never)	*Leptospira* spp. revaccination recommended by veterinarian (every year)	221/3,861	5.7	0.08	0.04–0.18
CDV and CPV revaccination recommended by veterinarian (less frequently than every 3 years)	CDV and CPV revaccination recommended by veterinarian (every 3 years)	54/3,871	1.4	0.21	0.04–0.72
*Leptospira* spp. revaccination recommended by veterinarian (unknown)	*Leptospira* spp. revaccination recommended by veterinarian (every year)	963/3,861	24.9	0.21	0.16–0.28
Owner’s knowledge about vaccination (poor)	Owner’s knowledge about vaccination (excellent)	88/3,864	2.3	0.24	0.10–0.53
*Leptospira* spp. revaccination recommended by veterinarian (every 3 years or less frequently)	*Leptospira* spp. revaccination recommended by veterinarian (every year)	152/3,861	3.9	0.27	0.15–0.47
*Leptospira* spp. revaccination recommended by veterinarian (only when needed)	*Leptospira* spp. revaccination recommended by veterinarian (every year)	146/3,861	3.8	0.29	0.14–0.62
Discouraged from vaccinations by VAAEs	No VAAEs in the past	826/3,860	21.4	0.35	0.26–0.49
Homeopathic practitioner very helpful as source of information about vaccination	Homeopathic practitioner as source of information about vaccination (not used)	336/3,746	9.0	0.37	0.24–0.57
Somewhat sceptical attitude of the respondent to vaccinations	Attitude of the respondent to vaccinations (very important to indispensable)	555/3,872	14.3	0.38	0.26–0.57
Age of the dog (≥10 years)	Age of the dog (15 months to 5 years)	633/3,881	16.3	0.39	0.28–0.53

OR = odds ratio; CI = confidence interval; AIC = Akaike information criterion; CPV = canine parvovirus; CDV = canine distemper virus; RV = rabies virus; VAAEs = vaccine-associated adverse effects.

Factors associated with the vaccination status of dogs resulted from a logistic regression after stepwise model selection based on the Akaike Information Criterion (AIC); p-values were omitted because of post-selection inference. The definition of the reference categories was made on a case-by-case basis; the option that was expected to reflect the response of most owners was chosen. Compared with this reference category, the expected probability of a variable in a dog with an ‘up-to-date vaccination status’ decreases with decreasing odds ratio (OR); these variables were therefore referred to as ‘negative’ influencing factors (Table 5).

## Discussion

The goals of the present study were to investigate vaccination status of dogs in Germany and owner compliance with vaccination. The results showed that less than half of the dogs had an ‘up-to-date vaccination status’ according to current vaccination guidelines; especially the vaccination rate against *Leptospira* spp. was poor. Furthermore, vaccination recommendations made by the veterinarian had a strong association with the vaccination status.

An ‘up-to-date vaccination status meant in the present study that vaccinations were required a minimum of every 3 years for CDV, CPV and RV and annually for *Leptospira* spp. This vaccine schedule is in accordance with vaccination guidelines and recommendations for core vaccinations in dogs in Germany [3]. In contrast to international guidelines (e.g., WSAVA) [4], leptospirosis vaccine has been considered a core vaccine in Germany since 2009 [5] and in other countries including the UK [23]. The main reason for including leptospirosis as a core vaccine is to prevent disease in individual dogs and to reduce urinary shedding of leptospires [24] and thus to protect humans. Protection of other animals, such as livestock, is also important as infected dogs could inadvertently wander onto pastures and increase the risk of infection in cattle by shedding *Leptospira* spp. [25]. The rate of leptospirosis in dogs had markedly increased in several European countries including Switzerland in the last 10 years [26] as well as in countries outside Europe [27]. A similar increase has also been seen in human medicine [28, 29] with about 1 million new infections every year worldwide. Leptospirosis is therefore one of the most important zoonotic diseases in the world [30]. Climate change is considered responsible for further increases in prevalence of infections because higher temperatures, increased precipitation and flooding enhance the survival time and spread of *Leptospira* spp. Progressive urbanisation and reduction of animal habitat also lead to a higher risk of infection [31, 32], which confirms the importance of including the leptospirosis component in a core vaccine program.

The duration of immunity (DOI) against RV varies from 1–3 years according to the vaccine manufacturer's specifications. In this study, however, the DOI was assumed to be 3 years regardless of the manufacturer's specifications because studies show that RV vaccination provides a DOI of at least 3 years [33]. Germany has been free of terrestrial rabies for many years. Rabies is currently still included in the core vaccines due to the national regulations [34]. Moreover, rabies is still present in large parts of the world [35, 36] and the majority of pet owners in Germany take their dog with them when travelling abroad (67%) [37], and rabies vaccination is required by law for crossing boarders in the European Union (EU) [34]. Rabies is a lethal zoonosis, and about 59,000 people die annually worldwide and a high number of cases go unreported [38]. In 99% of infections, the virus is transmitted to humans by a dog bite. Vaccination of dogs is therefore the most important measure to prevent human infections in endemic countries [39]. However, the core vaccine status of rabies vaccination might change in the future, as all EU countries will soon be declared ‘rabies-free’.

Vaccines against canine adenovirus (CAV-1 and CAV-2) are considered core vaccines according to some international guidelines [4]. However, CAV-1 causing infectious canine hepatitis has almost been eradicated from Western Europe [3, 40] and is no longer a core vaccine according to national guidelines [3]; CAV-2 only causes a very mild disease belonging to the CIRDC. Therefore, CAV vaccination was not considered part of an ‘up-to-date vaccination status’ in this study.

Based on the evaluation of the questionnaire and vaccination passports, owner compliance with vaccination guidelines was poor. Although this study is not necessarily representative of the whole dog population in Germany, it suggests that dogs might not be adequately protected. A comparison of information between the vaccination passports and owners’ responses revealed that owners had good knowledge on the vaccination status of their dogs. However, the number of dogs with an ‘up-to-date vaccination status’ was slightly higher in the group of participants who submitted vaccination passports than in the group that did not. This could have been because owners who sent in the vaccination passports of their dogs had a special interest in vaccination.

A study conducted in UK found that 78% of dogs had been vaccinated in the country, but information on used vaccines and vaccination intervals were missing, making comparison difficult [20]. Cat owner compliance with vaccination guidelines in Germany was shown to be considerably higher than dog owner compliance with 77.9% of cats having been currently vaccinated [18]. The most likely reason for the higher up-to-date vaccination status in cats is that all feline core vaccines provide a DOI of up to 3-year and thus vaccinations are commonly only performed every 3 years, while dogs need to be revaccinated annually for leptospirosis because of the short-term immunity afforded by this vaccine [3, 4]. In the present study, few owners stated that their dog had never been vaccinated, although it is possible that these participants were not aware of vaccinations the dog might have had before they became the owner.

Owner compliance with vaccination against *Leptospira* spp. was particularly poor, and only half of all dogs were up-to-date on this vaccine, which was concerning. Possible reasons for the poor compliance with vaccination against leptospirosis include scheduling issues and costs associated with annual vaccination and owner fear of VAAEs, especially in vaccines containing multiple serovars [41]. The German pharmacovigilance report showed increased VAAEs after leptospirosis vaccination that coincided with the market launch of multivalent vaccines against *Leptospira* spp. The VAAEs mainly manifested as an immunological hypersensitivity with oedema of the head, pruritus, vomiting and diarrhoea, as well as swelling and pain at the injection site. Approximately every 10th VAAE reported was an acute shock [42]. Another potential explanation for the low leptospirosis vaccination rate might have been that the dog owners were not aware of the seriousness of the disease in humans and in dogs. A Portuguese study found that dog owners lacked knowledge of the zoonotic potential of leptospirosis and its prevention [43]; this also likely applies to dog owners in Germany. In human medicine, inconsistent vaccination in children is associated with lack of parental knowledge of immunization [44], but vaccination rates can be increased by targeted educational measures [45, 46]. Sufficient consultation time and proper information from the veterinarian increases owner satisfaction and improves the client-veterinarian relationship, which can promote owner uptake of preventive measures [47–49]. A similar association was found in the present study; dogs belonging to owners who considered themselves poorly informed about vaccinations were less likely to be vaccinated than dogs of owners who considered themselves to be very well-informed.

Young dogs are more likely to have an ‘up-to-date vaccination status’ presumably because owners are aware of the importance of the primary vaccination series in establishing immunity. This is also evident in human medicine where young children have higher vaccination coverage than adults [50]. A possible reason for not vaccinating older dogs might be owner assumption that primary immunization leads to lifelong immunity. In the case of CDV and CPV, this presumption might actually be valid because studies showed long-term immunity in some dogs; e.g., antibodies were present in dogs 14 years after the use of a modified live CDV vaccine [51, 52]. However, it cannot be assumed that long-term immunity occurs in all dogs.

The higher probability that working dogs, such as police or rescue dogs, had an ‘up-to-date vaccination status’ was presumably due to vaccination guidelines within the respective organizations. The financial input for training these types of dogs could have also played a role in owner compliaance with vaccination guidelines. The increased vaccination rate in dogs travelling abroad in the previous 36 months was likely because of rabies regulations within the EU. The veterinarians had probably recommended other core vaccines when these dogs were presented for RV vaccination.

Surprisingly, almost half of the dog owners reported that annual CDV and CPV vaccination was recommended by their respective veterinarians. Dogs of these owners were more likely to have an ‘up-to-date vaccination status’, which shows that owners trust veterinary advice even though this advice is outdated. Revaccination against CDV and CPV, which involves modified live vaccines, should generally not be carried out more frequently than every 3 years [53]. This is recommended by both the WSAVA and German guidelines [3, 4] and is based on numerous studies on the DOI of these vaccines [51, 52, 54–56]. Even if the manufacturers specify a shorter DOI, the veterinarian should follow the recommendations of the guidelines, which equates to off-label use, because dogs should not be vaccinated more often than necessary [4]. Outdated vaccination recommendations could be attributable to ignorance on the part of the veterinarian and the fear of lost revenue with less frequent vaccinations. Dogs owned by participants who were unsure about their veterinarian's recommendations for RV revaccination had a higher probability of an‘up-to-date vaccination status’, but the reasons for this association are unknown.

The higher vaccination rate in dogs staying in boarding kennels (future and past stays) compared with dogs staying at home, can be attributed to the requirement of an up-to-date vaccination status. The association between owners who had received a tetanus vaccination within the last 10 years and the vaccination status of their dogs was of interest and suggests a link between the vaccination habits of owners and the vaccination status of their dogs. A human health survey in Portugal investigated factors associated with tetanus vaccination in immigrants. Higher household income and older age were negatively associated with tetanus vaccination rates, while living in sparsely populated areas was positively associated [57]. This could be due to the fact that people in rural areas are involved in agricultural work and therefore have more contact with soil in which the tetanus-causing pathogen is usually located. However, in the present study, the status on other vaccines (pertussis, influenza), age and sociodemographic background of the owners were not selected by the model and thus not associated with the vaccination status of the dogs.

Veterinary recommendations to owners on revaccinating dogs less frequently than every 12 months for leptospirosis had a strong ‘negative’ association with the vaccination status of the dog, especially when the advice was not to vaccinate against *Leptospira* spp. Such recommendations for leptospirosis vaccination are concerning and require further studies. It is not known why veterinarians consider vaccination against *Leptospira* spp. unimportant, but perhaps the disease is underdiagnosed contributing to their lack of awareness. It is possible that vaccination against leptospirosis is viewed critically by some veterinarians because >300 serovars in 24 serogroups are known [58], and yet vaccines in Germany are licensed for serovars of a maximum of 4 serogroups (Canicola, Icterohaemorrhagiae, Australis and Grippotyphosa) [59] and cross-immunity is restricted to serovars within a serogroup. Thus, despite vaccination, infection with *Leptospira* spp. cannot always be prevented in dogs [60]. However, the currently available canine leptospirosis vaccines have demonstrated to significantly reduce the number of leptospirosis cases in dogs, as demonstrated in Switzerland [61] and the vaccines contain the serogroups of the most common serovars also found in infected people in Germany (serovars of the serogroups Icterohaemorrhagiae, Canicola and Grippotyphosa) [62]. The canine vaccines not only prevent disease but also shedding of *Leptospira* spp. [24] and therefore reduce the zoonotic risk.

The finding that dogs were less likely to have an ‘up-to-date vaccination status’ when the veterinarian recommended a CDV/CPV revaccination interval of >3 years emphasizes the impact of veterinary recommendations on owners and the need to inform veterinarians of this issue. It is, however, possible that these dogs underwent testing for antibodies, which has been shown to be a useful tool for determining the necessity of revaccination [63, 64].

Occurrence of VAAEs in the past discouraged owners from having their dogs vaccinated and was ‘negatively’ associated with the vaccination status of the dog. Studies in cats and humans have shown that VAAEs have a negative effect on future vaccination decisions [18, 65]. Education about VAAEs appears to be important in allaying these concerns.

An association was found between the vaccination status of the dog and the owners' trust in the vaccination information provided by a homeopathic practitioner. This suggests that homeopathic practitioners, in contrast to veterinarians, are more likely to recommend alternative methods of disease prevention and discourage owners from having their dogs vaccinated, which is very concerning.

Compared with young dogs, an ‘up-to-date vaccination status’ occurred less frequently in older dogs, a finding that has also been reported in cats [18]. Owners of older animals might incorrectly assume that their pets are protected against infectious diseases because of long vaccination history or do not need vaccinations because infectious diseases do not occur in older cats and dogs. This assumption is supported by the finding that an ‘up-to-date vaccination status’ was more likely to be seen in dogs owned by participants who did not consider age as a very important factor in their decision on vaccination.

The association between the vaccination status of the dog and the residential area, the presence of children and the educational status of the owner was investigated, but the model did not select those variables. Interestingly, the model also did not select owner income or vaccination costs, which was in agreement with a study of cats in Germany [18]. These results indicate that pet owners in Germany are generally willing to pay for vaccination. This is in contrast to a cat study in the UK, where income and costs were selected by the model [2], although differences in the statistical model and vaccination guidelines used could have been the source of different findings. Many pets (57% dogs, 38% cats) have health insurance in the UK, but costs for vaccination are usually not covered [66]. Thus owners might not be accustomed or willing to pay for vaccination. In comparison, few cats (6%) and dogs (15%) are insured in Germany [67], and therefore owners are accustomed to paying for all veterinary costs. According to German veterinary fee regulations (GOT), the cost for vaccination of a dog (without including the cost of the vaccine) varies from 23.73 to 94.92 Euro, which is relatively inexpensive compared with treatment costs [68].

The limitations of the present study included validity of owners reporting an ‘up-to-date vaccination status’, which might not have always been correct. However, a sample of data on vaccination status reported by owners was checked by reviewing the vaccination passports and found to be in agreement. Nevertheless, it cannot be ruled out that owners, especially those who submitted vaccination passports, might have referred to this document while completing the survey, leading to an overestimation of owner knowledge on vaccination status. The definition of an 'up-to-date vaccination status' was based on RV revaccination every 3 years, although in Germany, RV vaccines with a shorter DOI (manufacturer’s specifications) are still available. However, it was assumed that most veterinarians follow the recommendation to vaccinate animals only as often as needed and therefore use vaccines with the longest possible DOI. Internet access was required for participation, which might have led to a certain bias, although the majority of the population in Germany (86%) uses the internet [69]. The survey was distributed via social media, and this might have attracted owners who were specifically interested in vaccination. However, a UK study showed that 78.6% of all pet owners use the internet as a source of information on health issues, especially social media and online forums [70]. A limitation of the statistical model was that post-selection inference is invalid and reference category selection can affect the results; classification into ‘positive’ or ‘negative’ depends directly on the choice of the reference category. Nevertheless, AIC is an established and common criterion for model selection as it balances goodness of fit with model complexity. Categorization into strongest ‘positive’ and ‘negative’ factors can result in strongly associated factors not being captured for discussion. However, all associated factors and their OR are listed in Table 1. Data similar to those reported in the present study do not exist in the current veterinary literature and therefore present an analysis that could serve as a model for further studies using causal inferences and binary variables.

## Conclusion

This is the first study that provides information on vaccination status of dogs and dog owner attitudes and compliance relating to vaccination in Germany. The results suggest that the vaccination status of dogs does not meet guidelines for core vaccines. Protection against leptospirosis appears to be particularly poor, which is concerning from a veterinary and human medical standpoint and requires urgent improvement. The association between veterinary recommendations and owner compliance was strong, providing an opportunity for veterinarians to improve the vaccination status of dogs. Further research on owner compliance with vaccination and the vaccination status of dogs would be valuable to validate the results.

## Supporting information

S1 Questionnaire Questionnaire in the original language German. (PDF)

S2 Questionnaire Questionnaire translated from German into English. (PDF)

S1 Table Demographic details of the participating dog owners. (DOCX)

S2 Table Characteristics of dogs owned by respondents participating in the web-based questionnaire (factors eliminated by the model). (DOCX)

S3 Table Past and future outings of dogs owned by respondents (factors eliminated by the model). (DOCX)

S4 Table Source of information regarding canine vaccination and attitude toward vaccination of the participating owners (factors eliminated by the model). (DOCX)

S5 Table Sociodemographic details of the participating owners (factors eliminated by the model). (DOCX)

S1 Dataset Raw data table of the 3,881 questionnaires answered by participating dog owners. (CSV)

S2 Dataset Calculation results by the Akaike information criterion. (CSV)

S3 Dataset Results of the vaccination passports and comparison with the respective questionnaires. (CSV)
