# Supplementary material for: Canine vaccination in Germany: A survey of owner attitudes and compliance
Source: PLoS One. 2020 Aug 27;15(8):e0238371. doi: 10.1371/journal.pone.0238371 (PMC7451643; doi:10.1371/journal.pone.0238371)
Supplement: S1 Questionnaire — (PDF) [file pone.0238371.s001.pdf]

Umfrage zur Impfung beim Hund

1.0 Informationsblatt

Sehr geehrte Hundebesitzer,

im Rahmen einer **Doktorarbeit an der Tierärztlichen Fakultät München** führen wir zu wissenschaftlichen Forschungszwecken eine **Umfrage zum Thema "Impfungen bei Hunden"** durch. Unser Ziel ist es, Ihre Meinung und Ihre Zufriedenheit bezüglich Aufklärung und Verbesserungsvorschläge in Bezug auf Impfmanagement beim Hund anhand dieses Fragebogens zu ermitteln. Neben unserem wissenschaftlichen Interesse hoffen wir, mit der Umfrage die Leistungen entsprechend zu verbessern und die Versorgung Ihres Hundes weiterhin auf einem hohen Niveau zu halten.

Ihre Angaben werden von uns statistisch und anonymisiert ausgewertet. Die Beantwortung des Fragebogens dauert circa 10 Minuten. Sollten beim Ausfüllen des Fragebogens Probleme auftreten, melden Sie sich bitte bei Simone Eschle (Simone.Eschle@campus.lmu.de).

Wenn Sie eine kostenlose Impfberatung erhalten möchten, können Sie am Ende des Fragebogens freiwillig Ihre E-Mail-Adresse angeben. Bitte senden Sie uns in diesem Fall auch eine Kopie des Impfausweises ihres Hundes (nähere Informationen am Ende der Umfrage). Für die Teilnahme an der Impfberatung ist es erforderlich, beiliegende Einwilligungserklärungen abzugeben. Die ausschließliche Teilnahme an der Umfrage, ohne Impfberatung, ist auch ohne Ihre Zustimmung in die Einwilligungserklärung möglich. In diesem Fall erfolgt die Datenerhebung anonym.

Von der Studie ausgeschlossen sind Personen unter 16 Jahren, sowie Tierärzte/Tierärztinnen!

Wir danken Ihnen für Ihre Teilnahme!

Katrin Hartmann Prof., Dr. med. vet., Dr. habil. Dipl. ECVIM (Internal Medicine) Klinikvorstand der Medizinischen Kleintierklinik Tierärztliche Fakultät Ludwig-Maximilians-Universität München

1.0 Informationsblatt [Fortsetzung]

Einwilligungserklärung (für Impfberatung)

Die Angabe Ihrer persönlichen Daten (Name und E-Mail-Adresse) erfolgt freiwillig und ist nicht verpflichtend. Ihre Daten werden gespeichert und anhand dieser Daten wird der zugesendete Impfausweis mit dem von Ihnen ausgefüllten Fragebogen verknüpft. Diese Verknüpfung dient zur Reevaluation der im Fragebogen gemachten Angaben anhand des Impfausweises. Das Ergebnis wird im Rahmen der Studie ausgewertet. Die von Ihnen angegebene E-Mail-Adresse wird zudem genutzt, um Ihnen das Ergebnis der durchgeführten Impfberatung zukommen zu lassen. Sobald diese Zuordnung erfolgt und/oder die Impfberatung durchgeführt wurde, werden Ihre persönlichen Daten gelöscht und Ihre Angaben anonymisiert. Im Weiteren verweisen wir auf die nachfolgende Datenschutzinformation gemäß Art. 13 DSGVO.

Sollten Sie der Einwilligungserklärung nicht zustimmen, können Sie dennoch anonym an der Umfrage teilnehmen, jedoch ist eine Teilnahme an der Impfberatung in diesem Fall ausgeschlossen.

1.1 *Ich bin damit einverstanden, dass meine persönlichen Daten (Name und E-Mail-Adresse) gespeichert und im Rahmen der Studie genutzt werden, um den von mir gesendeten Impfausweis des Hundes mit dem von mir ausgefüllten Fragebogen zu verknüpfen.*
Sobald diese Zuordnung erfolgt ist, werden die persönlichen Daten gelöscht.

Ja ☐

1.2 *Ich bin damit einverstanden, dass meine persönlichen Daten (Name und E-Mail-Adresse) gespeichert und genutzt werden, um den von mir gesendeten Impfausweis des Hundes im Rahmen der kostenlosen Impfberatung auszuwerten und dass die Impfberatung an die von mir angegebene E-Mail-Adresse gesendet werden darf. Sobald die Impfberatung erfolgt ist, werden die persönlichen Daten gelöscht.*

Ja ☐

1.0 Informationsblatt [Fortsetzung]

Datenschutzinformation gemäß Art. 13 DSGVO

1. Verantwortlich für die Datenverarbeitung
Ludwig-Maximilians-Universität München, gesetzlich vertreten durch den Präsidenten, Herrn Prof. Dr. Bernd Huber, Geschwister-Scholl-Platz 1, 80539 München

Zuständige Organisationseinheit bzw. Dienststelle für die Datenverarbeitung:
Ludwig-Maximilians-Universität München, Medizinische Kleintierklinik, Veterinärstraße 13, 80539 München, Telefon: 089 21802650, E-Mail: Simone.Eschle@campus.lmu.de

2. Kontaktdaten des behördlichen Datenschutzbeauftragten
Der behördliche Datenschutzbeauftragte der Ludwig-Maximilians-Universität München, Geschwister-Scholl-Platz 1, 80539 München, Telefon: +49 (0) 89 2180 - 2414, Telefax: +49 (0) 89 2180 - 2985, E-Mail: datenschutz@lmu.de

3. Art der verarbeiteten Daten und Zweck der Datenverarbeitung
Zweck der Datenverarbeitung ist Erstellung einer Studie zum Thema „Impfungen beim Hund". Eine Datenverarbeitung zu anderen als zu den angegebenen bzw. gesetzlich zugelassenen Zwecken erfolgt nicht.
Wir verarbeiten nur die personenbezogenen Daten, die Sie uns zur Verfügung gestellt haben (Art. 4 Abs. 2 BayDSG).

4. Rechtsgrundlagen der Datenverarbeitung
Wir verarbeiten Ihre Daten im Einklang mit und auf Basis der Datenschutz-Grundverordnung, des Bayerischen Datenschutzgesetzes und der sonstigen anwendbaren Datenschutzbestimmungen. Die Datenverarbeitung erfolgt vorliegend auf Grundlage Ihrer Einwilligung gemäß Art. 6 Abs. 1 lit. a DSGVO.

5. Automatisierte Entscheidungsfindung
Ihre Daten werden nicht in rein automatisierten Verarbeitungsprozessen zur Herbeiführung einer Entscheidung verarbeitet.

6. Weitergabe der Daten an Dritte
Die Verarbeitung erfolgt auf internen Datenverarbeitungsanlagen. Innerhalb der Ludwig-Maximilians-Universität München erhalten nur diejenigen Personen bzw. Bereiche Ihre personenbezogenen Daten, die diese für die Erfüllung der genannten Verarbeitungszwecke benötigen. Ihre Daten werden nicht an Dritte oder in ein Drittland weitergegeben.

7. Dauer der Datenspeicherung
Die Dauer der Speicherung der Daten bestimmt sich nach den gesetzlichen Vorschriften. Die Daten werden gelöscht, sobald und soweit sie für die genannten Verarbeitungszwecke nicht mehr erforderlich sind und aufgrund gesetzlicher Vorschriften nicht mehr benötigt werden. Aus wissenschaftlichen Gründen kann eine Speicherung bis zu 10 Jahren notwendig sein.

8. Ihre Rechte
Sie haben das Recht, Auskunft über die zu Ihrer Person gespeicherten Daten zu erhalten (Art. 15 DSGVO). Sollten unrichtige personenbezogene Daten verarbeitet werden, steht Ihnen ein Recht auf Berichtigung zu (Art. 16 DSGVO). Zudem haben Sie das Recht auf Löschung (Art. 17 DSGVO), Beschränkung (Art. 18 DSGVO) und Widerspruch (Art. 21 DSGVO). Diese Rechte sind unter den Voraussetzungen des Art. 25 Abs. 4 BayDSG beschränkt, soweit die Inanspruchnahme dieser Rechte voraussichtlich die Verwirklichung der wissenschaftlichen Forschungszwecke unmöglich macht oder ernsthaft beeinträchtigt und diese Beschränkung für die Erfüllung der Forschungszwecke notwendig ist.

Sie haben das Recht auf Widerruf für die Zukunft. Dieser ist ohne Angabe von Gründen zu richten an Simone.Eschle@campus-lmu.de. Nach erfolgtem Widerruf werden Ihre Daten sofort gelöscht. Die Rechtmäßigkeit, der aufgrund der Einwilligung bis zum Widerruf erfolgten Datenverarbeitung, wird durch diesen nicht berührt.

Sollten Sie von Ihren Rechten Gebrauch machen, prüft die für die Datenverarbeitung verantwortliche Stelle, ob die gesetzlichen Voraussetzungen hierfür erfüllt sind und trifft dann die erforderlichen Maßnahmen. Bitte wenden Sie sich bei Fragen oder Beschwerden an die unter Ziffer 1 genannte Dienststelle. Darüber hinaus steht Ihnen gemäß Art. 77 DSGVO das Recht zur Beschwerde bei einer Datenschutzaufsichtsbehörde zu. Die für die Ludwig-Maximilians-Universität München zuständige Aufsichtsbehörde ist der Bayerische Landesbeauftragte für den Datenschutz (Postfach 22 12 19, 80502 München, Telefon: 089 212672-0, Telefax: 089 212672-50, E-Mail: poststelle@datenschutz-bayern.de, Internet: www.datenschutz-bayern.de).
Bitte setzen Sie sich in jedem Fall zunächst mit der unter Ziffer 1 genannten Dienststelle in Verbindung, um Ihr Anliegen zu klären.

2.0 Abschnitt A

Abschnitt A

Der erste Abschnitt bezieht sich auf Informationen über Ihren Hund oder Ihre Hunde.

Wenn Sie mehr als einen Hund besitzen, beantworten Sie die folgenden Fragen für den Hund, dessen Name nach alphabetischer Reihenfolge als ERSTES vorkommt.

3.0

3.1 **Wie viele Hunde besitzen Sie?**
- ☐ Einen Hund
- ☐ Zwei Hunde
- ☐ Drei Hunde
- ☐ Vier oder mehr Hunde

3.2 **Wie alt ist ihr Hund?**
- ☐ Unter 8 Wochen
- ☐ 8 Wochen bis 16 Wochen
- ☐ 16 Wochen bis 15 Monate
- ☐ 15 Monate bis 5 Jahre
- ☐ 5 Jahre bis 10 Jahre
- ☐ 10 Jahre oder älter
- ☐ Das Alter ist mir nicht bekannt

3.3 **Wie alt war Ihr Hund, als er von Ihnen aufgenommen wurde?**
- ☐ Unter 8 Wochen
- ☐ 8 Wochen bis 16 Wochen
- ☐ 16 Wochen bis 15 Monate
- ☐ 15 Monate bis 5 Jahre
- ☐ 5 Jahre bis 10 Jahre
- ☐ 10 Jahre oder älter
- ☐ Das Alter ist nicht bekannt

3.4 **Woher haben Sie Ihren Hund?**
- ☐ Aus einem Tierheim oder einer Tierschutzorganisation- aus dem Ausland
- ☐ Aus einem Tierheim oder einer Tierschutzorganisation- aus Deutschland
- ☐ Vom anerkannten Züchter eines Zuchtverbandes
- ☐ Von einer Privat- oder Hobbyzucht
- ☐ Über eine Zeitungsanzeige
- ☐ Über das Internet
- ☐ Von Freunden, Verwandten oder Nachbarn
- ☐ Sonstiges

3.5 **Ist Ihr Hund ein Rassehund?**
- ☐ Ja
- ☐ Nein
- ☐ Das weiß ich nicht

3.6 **Wenn Ihr Hund reinrassig ist, welcher Rasse gehört er an?**

____

3.7 **Wie gehen Sie mit Ihrem Hund Gassi?**
- ☐ Mein Hund ist ausschließlich an der Leine
- ☐ Mein Hund läuft ausschließlich frei
- ☐ Beides

MUSTER

3.0 [Fortsetzung]

3.8 **Geht Ihr Hund im Sommer baden?**
- ☐ Nein, er ist sehr wasserscheu
- ☐ Ja, aber selten (weniger als 1x wöchentlich)
- ☐ Ja, häufig

3.9 **Wenn ihr Hund baden geht, wie geht er ins Wasser?**
- ☐ Nur mit den Pfoten
- ☐ Er schwimmt vollständig

3.10 **Welchem Haltungszweck dient ihr Hund?** (mehrere Antwortmöglickeiten)
- ☐ Familienhund
- ☐ Zuchthund
- ☐ Jagdlicher Gebrauch
- ☐ Für sportliche Aktivitäten
- ☐ Wach- oder Schutzhund
- ☐ Diensthund (z.B. Rettungshund, Polizeihund)
- ☐ Assistenzhund (z.B. Blindenhund, Diabetikerwarnhund)

3.11 **Bitte kreuzen Sie an, ob Ihr Hund in den letzten <u>12 Monaten</u> eine oder mehrere der folgenden Möglichkeiten besucht hat.**
- ☐ Hundepension oder Hundesitter
- ☐ Hundeausstellung oder Hundeshow
- ☐ Hundeschule
- ☐ Hundesport
- ☐ Keines der oben genannten

3.12 **Bitte kreuzen Sie an, ob Ihr Hund in den letzten <u>24 Monaten</u> eine oder mehrere der folgenden Möglichkeiten besucht hat.**
- ☐ Hundepension oder Hundesitter
- ☐ Hundeausstellung oder Hundeshow
- ☐ Hundeschule
- ☐ Hundesport
- ☐ Keines der oben genannten

3.13 **Bitte kreuzen Sie an, ob Ihr Hund in den letzten <u>36 Monaten</u> eine oder mehrere der folgenden Möglichkeiten besucht hat.**
- ☐ Hundepension oder Hundesitter
- ☐ Hundeausstellung oder Hundeshow
- ☐ Hundeschule
- ☐ Hundesport
- ☐ Keines der oben genannten

3.14 **War Ihr Hund in den letzten Monaten im Ausland?**
- ☐ Ja, in den letzten 12 Monaten
- ☐ Ja, in den letzten 24 Monaten
- ☐ Ja, in den letzten 36 Monaten
- ☐ Ja, aber länger als 36 Monate her
- ☐ Nein, er war innerhalb der letzten 36 Monate nicht im Ausland

3.15 **Bekommt Ihr Hund derzeit Medikamente zur Behandlung einer Erkrankung?**
- ☐ Ja
- ☐ Nein
- ☐ Unbekannt

3.16 **Wenn ja, welches Medikament bekommt Ihr Hund gegen welche Erkrankung verabreicht?**

MUSTER

4.0 Planen Sie, Ihren Hund in den kommenden 36 Monaten zu den folgenden Möglichkeiten mitzunehmen?

		Ja	Nein	Vielleicht
4.1	Hundepension	☐	☐	☐
4.2	Hundeausstellung oder Hundeshow	☐	☐	☐
4.3	Hundeschule	☐	☐	☐
4.4	Hundesport	☐	☐	☐

5.0

5.1 **Planen Sie, Ihren Hund in den kommenden 36 Monaten mit ins Ausland zu nehmen?**
☐ Ja, innerhalb der EU ☐ Ja, außerhalb der EU ☐ Nein
☐ Unsicher

5.2 **Wenn ja, in welches Land reisen Sie?**

5.3 **Ist Ihr Hund schon einmal geimpft worden?** ☐ Ja ☐ Nein

6.0 Bitte geben Sie eine Einschätzung ab, welche Wichtigkeit die unten angegebenen Impfungen Ihrer Meinung nach haben

		Sehr wichtig	Wichtig	Weniger wichtig	Unwichtig	Mir nicht bekannt
6.1	Leptospirose	☐	☐	☐	☐	☐
6.2	Zwingerhustenkomplex	☐	☐	☐	☐	☐
6.3	Leishmaniose	☐	☐	☐	☐	☐
6.4	Hepatitis contagiosa canis	☐	☐	☐	☐	☐
6.5	Staupe	☐	☐	☐	☐	☐
6.6	Parvovirose (Hundeseuche)	☐	☐	☐	☐	☐
6.7	Canines Herpesvirus	☐	☐	☐	☐	☐
6.8	Tollwut	☐	☐	☐	☐	☐
6.9	Hautpilze	☐	☐	☐	☐	☐
6.10	Borreliose	☐	☐	☐	☐	☐

7.0

7.1 **Wann, wenn überhaupt, hat Ihr Hund seine letzte Leptospirose Impfung oder Boosterung (Auffrischung) erhalten?**
☐ Innerhalb des letzten Jahres ☐ Vor mehr als 1 Jahr bis 3 Jahren ☐ Vor mehr als 3 Jahren
☐ Nie ☐ Weiß ich nicht

MUSTER

7.0 [Fortsetzung]

7.2 **Wann, wenn überhaupt, hat Ihr Hund seine letzte Tollwut Impfung oder Boosterung (Auffrischung) erhalten?**

- ☐ Innerhalb des letzten Jahres
- ☐ Vor mehr als 1 Jahr bis 3 Jahren
- ☐ Vor mehr als 3 Jahren
- ☐ Nie
- ☐ Weiß ich nicht

7.3 **Wann, wenn überhaupt, hat Ihr Hund seine letzte Staupe, Hepatitis contagiosa canis, Parvovirose Impfung oder Boosterung (Auffrischung) erhalten?**

- ☐ Innerhalb des letzten Jahres
- ☐ Vor mehr als 1 Jahr bis 3 Jahren
- ☐ Vor mehr als 3 Jahren
- ☐ Nie
- ☐ Weiß ich nicht

7.4 **In welchen Abständen wird Ihr Hund Leptospirose geimpft?**

- ☐ Jedes Jahr
- ☐ Alle 2 Jahre
- ☐ Alle 3 Jahre
- ☐ Weniger oft als alle 3 Jahre
- ☐ Nie
- ☐ Weiß ich nicht

7.5 **In welchen Abständen wird Ihr Hund Tollwut geimpft?**

- ☐ Jedes Jahr
- ☐ Alle 2 Jahre
- ☐ Alle 3 Jahre
- ☐ Weniger oft als alle 3 Jahre
- ☐ Nie
- ☐ Weiß ich nicht

7.6 **In welchen Abständen wird Ihr Hund Staupe, Hepatitis contagiosa canis, Parvovirose geimpft?**

- ☐ Jedes Jahr
- ☐ Alle 2 Jahre
- ☐ Alle 3 Jahre
- ☐ Weniger oft als alle 3 Jahre
- ☐ Nie
- ☐ Weiß ich nicht

8.0 Bitte kreuzen Sie jeweils an, ob Sie die folgenden Möglichkeiten bei der Informierung über Impfungen als hilfreich empfunden haben:

		Sehr hilfreich	Hilfreich	Nicht hilfreich	Quelle nicht genutzt
8.1	Tierarzt/ärztin oder Tierarzthelfer/in	☐	☐	☐	☐
8.2	Tierheilpraktiker/in	☐	☐	☐	☐
8.3	Internet	☐	☐	☐	☐
8.4	Bücher, Zeitschriften	☐	☐	☐	☐
8.5	Freunde, Verwandte, Kollegen	☐	☐	☐	☐
8.6	Züchter	☐	☐	☐	☐
8.7	Hundeschule	☐	☐	☐	☐
8.8	Zoofachgeschäft	☐	☐	☐	☐

8.9 Sonstiges:

[ ]

MUSTER

9.0

9.1 **Wie gut fühlen Sie sich selbst über Impfungen beim Hund informiert?**
- ☐ Sehr gut, ich denke ich bin über alles wichtige aufgeklärt
- ☐ Mittelmäßig, ich denke ich weiß einiges aber es könnte noch mehr sein
- ☐ Eher schlecht, so richtig viel weiß ich nicht über die Impfungen
- ☐ Schlecht, ich kenne mich mit den Impfungen gar nicht aus

9.2 **Wünschen Sie sich mehr Aufklärung und Informationen über Impfungen von Ihrem Tierarzt?**
- ☐ Ja
- ☐ Nein

9.3 **Zu welchen Impfabständen hat Ihr Haustierarzt Ihnen bezüglich <u>Leptospirose</u> geraten?**
- ☐ Jedes Jahr
- ☐ Alle 2 Jahre
- ☐ Alle 3 Jahre
- ☐ Seltener als 3 Jahre
- ☐ Nur bei Bedarf, nach Antikörpermessung
- ☐ Nie
- ☐ Weiß ich nicht

9.4 **Zu welchen Impfabständen hat Ihr Haustierarzt Ihnen bezüglich <u>Tollwut</u> geraten?**
- ☐ Jedes Jahr
- ☐ Alle 2 Jahre
- ☐ Alle 3 Jahre
- ☐ Seltener als 3 Jahre
- ☐ Nur bei Bedarf, nach Antikörpermessung
- ☐ Nie
- ☐ Weiß ich nicht

9.5 **Zu welchen Impfabständen hat Ihr Haustierarzt Ihnen bezüglich <u>Staupe, Hepatitis contagiosa canis, Parvovirose</u> geraten?**
- ☐ Jedes Jahr
- ☐ Alle 2 Jahre
- ☐ Alle 3 Jahre
- ☐ Seltener als 3 Jahre
- ☐ Nur bei Bedarf, nach Antikörpermessung
- ☐ Nie
- ☐ Weiß ich nicht

10.0 Bitte bewerten Sie, wie wichtig Ihnen die folgenden Punkte bei der Entscheidung sind, ob Sie Ihren Hund impfen lassen oder nicht *(1= unwichtig und 5= sehr wichtig)*:

		1	2	3	4	5
10.1	Kosten	☐	☐	☐	☐	☐
10.2	Nebenwirkungen	☐	☐	☐	☐	☐
10.3	Eine stressvolle Erfahrung für Ihren Hund	☐	☐	☐	☐	☐
10.4	Die Impfung wird für eine Impfbescheinigung benötigt (z.B. für Hundepesion, Hundeshow, etc.)	☐	☐	☐	☐	☐
10.5	Die Impfung wird für eine Auslandsreise benötigt	☐	☐	☐	☐	☐
10.6	Tierärztliche Beratung	☐	☐	☐	☐	☐
10.7	Die Wahrscheinlichkeit, dass Ihr Hund an dieser Krankheit erkranken kann	☐	☐	☐	☐	☐
10.8	Die Wirksamkeit der Impfung	☐	☐	☐	☐	☐
10.9	Die Schwere der Krankheiten, gegen die die Impfung schützt	☐	☐	☐	☐	☐
10.10	Zeitaufwand und Unannehmlichkeiten	☐	☐	☐	☐	☐
10.11	Das Alter des Hundes	☐	☐	☐	☐	☐

MUSTER

11.0

11.1 **Welcher der folgenden Punkte würde Sie von einem Routinetermin für eine Impfung bei Ihrem Tierarzt abhalten?** (Mehrfachnennungen möglich)

- ☐ Öffnungszeiten
- ☐ Zeitaufwand
- ☐ Stress beim Besuch/ Transport
- ☐ Kosten
- ☐ Potentielle Nebenwirkungen
- ☐ Keine der oben genannten Möglichkeiten
- ☐ Nicht zutreffend, da der Hund nicht geimpft wird
- ☐ Sonstiges

11.2 Bitte nennen Sie Ihre sonstigen Gründe:

12. Abschnitt B

Abschnitt B

In diesem Abschnitt sollten Sie alle Hunde berücksichtigen, die Sie besitzen oder besessen haben.

13.0 Bitte geben Sie an, ob einer Ihrer Hunde an folgenden Infektionskrankheiten erkrankt ist oder war: Staupe, Parvovirose (Hundeseuche), Leptospirose, Tollwut, Hepatitis contagiosa canis.

	Ja	Unsicher	Nein
13.1 Ich habe/ hatte einen **ungeimpften** Hund mit einer dieser Erkrankungen	☐	☐	☐
13.2 Ich habe/ hatte einen **geimpften** Hund mit einer dieser Erkrankungen	☐	☐	☐

13.3 **Wenn ja, geben Sie bitte an, an welcher dieser Krankheiten Ihr ungeimpfter Hund erkrankt ist oder war:**

13.4 **Wenn ja, geben Sie bitte an, an welcher dieser Krankheiten Ihr geimpfter Hund erkrankt ist oder war:**

14.0

14.1 **Hat eine Impfung jemals bei Ihrem Hund Nebenwirkungen verursacht?** ☐ Ja ☐ Nein

14.2 **Welche Art von Nebenwirkungen hatte Ihr Hund?**

- ☐ Mattigkeit
- ☐ Fieber
- ☐ Lahmheit
- ☐ Sonstiges
- ☐ Appetitlosigkeit
- ☐ Erbrechen
- ☐ Allergische Reaktion (innerhalb von 24 Stunden)
- ☐ Reaktionen an der Injektionsstelle
- ☐ Durchfall
- ☐ Immunmediierte Erkrankung

14.3 **Welche immunmediierte Erkrankung wurde durch die Impfung hervorgerufen?**

14.0 [Fortsetzung]

14.4 **Welche sonstigen Nebenwirkungen haben sich bei Ihrem Hund gezeigt?**

14.5 **Diese Nebenwirkungen waren:**

- ☐ Unbedeutend und selten
- ☐ Unbedeutend und oft
- ☐ Schwerwiegend und selten
- ☐ Schwerwiegend und oft

14.6 **Haben diese Nebenwirkungen Sie von weiteren Impfungen abgehalten?**

- ☐ Ja
- ☐ Nein

15.0 Abschnitt C

Abschnitt C

Der letzte Abschnitt enthält Fragen zu Ihnen und Ihrem Haushalt. Diese Informationen helfen uns, Meinungen über "Impfungen beim Hund" mit dem Querschnitt der deutschen Haushalte zu vergleichen. Ihre Daten werden nicht für andere Zwecke verwendet. Wenn es Fragen gibt, die Sie nicht beantworten möchten, lassen Sie die Antwortmöglichkeiten leer und gehen Sie zur nächsten Frage weiter.

15.1 **Welches Geschlecht haben Sie?**
- ☐ weiblich
- ☐ männlich

15.2 **In welchem Bundesland leben Sie?**
- ☐ Baden- Württemberg
- ☐ Bayern
- ☐ Berlin
- ☐ Brandenburg
- ☐ Bremen
- ☐ Hamburg
- ☐ Hessen
- ☐ Mecklenburg- Vorpommern
- ☐ Niedersachsen
- ☐ Nordrhein- Westfalen
- ☐ Rheinland- Pfalz
- ☐ Saarland
- ☐ Sachsen
- ☐ Sachsen-Anhalt
- ☐ Schleswig- Holstein
- ☐ Thüringen

15.3 **Ihr Wohngebiet liegt:**
- ☐ Im ländlichen Bereich (unter 50.000 Einwohner)
- ☐ Im Einzugsbereich einer Stadt (50.000 bis 500.000 Einwohner)
- ☐ Im Einzugsbereich einer Großstadt (ab 500.000 Einwohner)

15.4 **Was ist der höchste Bildungsgrad in Ihrem Haushalt?**
- ☐ Hauptschulab-schluss
- ☐ Realschulabschluss
- ☐ Abitur
- ☐ Hochschulabs-chluss
- ☐ Kein Abschluss

15.5 **Ihr jährliches Einkommen beträgt:**
- ☐ unter 10.000 Euro
- ☐ zwischen 10.000 Euro und 25.000 Euro
- ☐ zwischen 25.000 Euro und 50.000 Euro
- ☐ zwischen 50.000 Euro und 75.000 Euro
- ☐ zwischen 75.000 Euro und 100.000 Euro
- ☐ über 100.000 Euro

15.6 **Haben Sie Kinder?**
- ☐ Ja
- ☐ Nein

15.7 **Wieviele Kinder unter 18 Jahren leben in Ihrem Haushalt?**
- ☐ Keine
- ☐ 1
- ☐ 2
- ☐ 3
- ☐ 4 oder mehr

15.8 **Wurde Ihr jüngstes Kind innerhalb des ersten Lebensjahres geimpft?**
- ☐ Ja
- ☐ Nein

15.0 **Abschnitt C** [Fortsetzung]

15.9 **Wie alt sind Sie?**

15.10 **Wie sind Sie selbst Impfungen gegenüber eingestellt?**
- ☐ Ich finde Impfungen als Krankheitsprä- vention sehr wichtig bis unverzichtbar
- ☐ Ich finde Impfungen sinnvoll, wäge aber Vor-und Nachteile gründlich ab
- ☐ Ich bin Impfungen gegenüber eher skeptisch
- ☐ Ich bin grundsätzlich gegen Impfungen aller Art

15.11 **Ich bin generell gegen Impfungen aus folgenden Gründen (Mehrfachnennungen möglich):**
- ☐ Impfungen sind überflüssig und unnötig
- ☐ Impfungen sind gesundheitsschädlich, sie schwächen das Immunsystem
- ☐ Impfungen können andere Erkrankungen auslösen
- ☐ Impfungen dienen nur der Bereicherung von Ärzten und Pharmaindustrie
- ☐ Sonstige Gründe

15.12 **Bitte nennen Sie Ihre sonstigen Gründe:**

15.13 **Sind Sie selbst die letzten 10 Jahre <u>Tetanus</u> geimpft worden?** ☐ Ja ☐ Nein ☐ Unbekannt

15.14 **Sind Sie selbst die letzten 10 Jahre <u>Diphtherie</u> geimpft worden?** ☐ Ja ☐ Nein ☐ Unbekannt

15.15 **Sind Sie selbst die letzten 10 Jahre <u>Keuchhusten</u> geimpft worden?** ☐ Ja ☐ Nein ☐ Unbekannt

15.16 **Sind Sie selbst innerhalb des letzten Jahres <u>Influenza</u> geimpft worden?** ☐ Ja ☐ Nein ☐ Unbekannt

16.0 Abschnitt D- Impfberatung

Impfberatung: FREIWILLIG und nicht verpflichtend

Sollten Sie eine kostenlose Impfberatung durch die Medizinische Kleintierklinik der LMU wünschen, senden Sie uns bitte als Foto oder Scan die Seiten des Impfausweises mit allen Impfungen, sowie die Seite mit den Daten (wichtig ist das Geburtsdatum) Ihres Hundes (über welchen Sie auch die Umfrage beantwortet haben) per Email an **Simone.Eschle@campus.lmu.de** und tragen Sie im folgenden Textfeld bitte Ihre eigene Email-Adresse ein:

16.1 **Um eine kostenlose Impfberatung zu erhalten, tragen Sie bitte hier Ihre Email-Adresse ein *(nicht verpflichtend):***

17.0 Abschnitt E

17.1 **Haben Sie Verbesserungsvorschläge, Wünsche oder Anregungen?**

Vielen Dank für Ihre Mitarbeit!
