# Supplementary material for: Canine vaccination in Germany: A survey of owner attitudes and compliance
Source: PLoS One. 2020 Aug 27;15(8):e0238371. doi: 10.1371/journal.pone.0238371 (PMC7451643; doi:10.1371/journal.pone.0238371)
Supplement: S2 Questionnaire — (PDF) [file pone.0238371.s002.pdf]

Survey on dogs' vaccinations

1.0 Information sheet

Dear dog owners,

within the framework of a doctoral thesis at the Faculty of Veterinary Medicine at the LMU in Munich, we are conducting a scientific survey with research subject "dogs' vaccinations". Purpose is to determine your opinion, attitude and satisfaction regarding vaccination management of dogs. Moreover, by means of this questionnaire we are aiming to examine your view on vaccination information management in general as well as suggestions for improvement. Complementary to our scientific approach, we trust that this research will help us to improve our services accordingly and to maintain the high level of care quality of your dog.

Your information and data will be evaluated anonymously and statistically. It takes around 10 minutes to answer the questionnaire. Should you encounter any problems when filling in the survey, please contact Simone Eschle (Simone.Eschle@campus.lmu.de).

If you would like to receive a free vaccination consultation in return for your efforts, you can voluntarily enter your e-mail address at the end of the questionnaire. In this case, please submit the enclosed declarations of consent separately. Furthermore, please also send us a copy of your dog's vaccination passport (you can find more information on this at the end of the questionnaire). A mere participation in the survey, without vaccination advice, is also possible without your consent in the declaration of consent. In this case the data collection will be anonymous.

Persons under 16 years of age and veterinarians are excluded from the study.

Thank you for your participation!

Katrin Hartmann Prof., Dr. med. vet., Dr. habil. Dipl. ECVIM (Internal Medicine) Clinical Director of the Medical Small Animal Clinic Veterinary Faculty Ludwig-Maximilians-University Munich

1.0 Information sheet [continued]

Declaration of consent (for vaccination advice)

The provision of your personal data (name and e-mail address) is voluntary and not obligatory. Your data will be stored. Based on this the sent vaccination passport will be linked to the questionnaire you have filled in. This link is used to re-evaluate the information provided in the questionnaire on the basis of the vaccination passport. The result will be evaluated within the framework of the study. The provided e-mail address will also be used to send you the results of the vaccination consultation. As soon as this allocation is made and/or the vaccination advice has been carried out, your personal data will be deleted and your details will be made anonymous. Furthermore, we refer to the following data protection information in accordance with article 13 General Data Protection Regulation (GDPR).

If you do not agree to the declaration of consent, you can surely still participate anonymously in the survey. In this case participation in the vaccination advice is excluded.

1.1 *I agree that my personal data (name and e-mail address) will be stored and used within the scope of the study in order to link the dog's vaccination card sent by me with the questionnaire I have completed.*
As soon as this assignment has been made, the personal data will be deleted.

Yes ☐

1.2 *I agree that my personal data (name and e-mail address) will be stored and used to evaluate the dogs' vaccination passport sent by me in the context of the free vaccination advice and that the vaccination advice may be sent to the e-mail address I have provided. As soon as the vaccination advice has been given, the personal data will be deleted.*

Yes ☐

1.0 Information sheet [continued]

Data protection information according to Art. 13 DSGVO

1. Responsible for data processing
Ludwig-Maximilians-University Munich, legally represented by the president, Prof. Dr. Bernd Huber, Geschwister-Scholl-Platz 1, 80539 Munich

Responsible organisational unit or department for data processing:
Ludwig-Maximilians-University Munich, Medical Clinic for Small Animals, Veterinärstraße 13, 80539 Munich, Germany, telephone: 089 21802650, e-mail: Simone.Eschle@campus.lmu.de

2. Contact details of the official data protection officer
The official data protection officer of the Ludwig-Maximilians-University Munich, Geschwister-Scholl-Platz 1, 80539 Munich, Germany, telephone: +49 (0) 89 2180 - 2414, fax: +49 (0) 89 2180 - 2985, e-mail: datenschutz@lmu.de

3. Type of data processed and purpose of data processing
The purpose of the data processing is the preparation of a study on the topic "vaccinations in dogs". No data processing will be carried out for other purposes than those stated or permitted by law.
We only process the personal data that you have made available to us (Art. 4 para. 2 BayDSG).

4. Legal basis of the data processing
We process your data in accordance with and on the basis of the Basic Data Protection Ordinance, the Bavarian Data Protection Act and other applicable data protection regulations. The data processing in the present case is based on your consent in accordance with Art. 6 Para. 1 lit. a DSGVO.

5. Automated decision making
Your data will not be processed in purely automated processes to reach a decision.

6. Transfer of data to third parties
The processing is carried out on internal data processing equipment. Within the Ludwig-Maximilians-University Munich, only those persons or departments that need your personal data to fulfil the aforementioned processing purposes will receive them. Your data will not be passed on to third parties or to a third country.

7. Duration of data storage
The duration of the storage of the data is determined by the legal regulations. The data will be deleted as soon as and to the extent that they are no longer required for the aforementioned processing purposes and are no longer needed due to legal regulations. For scientific reasons, storage may be necessary for up to 10 years.

8. Your rights
You have the right to receive information about the data stored about you (Art. 15 DSGVO). Should incorrect personal data be processed, you have the right to have it corrected (Art. 16 DSGVO). You also have the right to deletion (Art. 17 DSGVO), restriction (Art. 18 DSGVO) and objection (Art. 21 DSGVO). These rights are limited under the conditions of Art. 25 para. 4 BayDSG, insofar as the exercise of these rights is likely to make the realisation of the scientific research purposes impossible or seriously impair them and this limitation is necessary for the fulfilment of the research purposes.

You have the right of revocation for the future. This is to be sent without giving reasons to Simone.Eschle@campus-lmu.de. After revocation your data will be deleted immediately. This does not affect the lawfulness of the data processing that took place on the basis of the consent until the revocation.

Should you make use of your rights, the body responsible for data processing will check whether the legal requirements are met and will then take the necessary measures. If you have any questions or complaints, please contact the service mentioned under point 1. In addition, pursuant to Art. 77 DSGVO, you have the right to complain to a data protection supervisory authority. The supervisory authority responsible for the Ludwig-Maximilians-University Munich is the Bavarian State Commissioner for Data Protection (Postfach 22 12 19, 80502 Munich, Telephone: 089 212672-0, Fax: 089 212672-50, E-Mail: poststelle@datenschutz-bayern.de, Internet: www.datenschutz-bayern.de).
In any case, please contact the office mentioned under point 1 first to clarify your request.

2.0 Section A

Section A

The first section relates to information about your dog or dogs.

If you own more than one dog, answer the following questions for the dog whose name appears in alphabetical order as FIRST.

3.0

3.1 How many dogs do you own?

- ☐ One dog
- ☐ Two dogs
- ☐ Three dogs
- ☐ Four or more dogs

3.2 How old is your dog?

- ☐ Under 8 weeks
- ☐ 8 weeks to 16 weeks
- ☐ 16 weeks to 15 moths
- ☐ 15 months to 5 years
- ☐ 5 years to 10 years
- ☐ 10 years or older
- ☐ I don't know the age

3.3 How old was your dog when you took him?

- ☐ Under 8 weeks
- ☐ 8 weeks to 16 weeks
- ☐ 16 weeks to 15 moths
- ☐ 15 months to 5 years
- ☐ 5 years to 10 years
- ☐ 10 years or older
- ☐ The age is unknown

3.4 From where did you get your dog?

- ☐ From an animal shelter or animal charity- from abroad
- ☐ From an animal shelter or animal charity- from Germany
- ☐ From the recognized breeder of a breeding association
- ☐ From a private or hobby breeder
- ☐ Via a newspaper advertisement
- ☐ Via the internet
- ☐ From friends, relatives or neighbours
- ☐ Others

3.5 Is your dog a purebred dog?

- ☐ Yes
- ☐ No
- ☐ Unknown

3.6 If your dog is purebred, what breed does the dog belong to?

____

3.7 How do you take your dog for a walk?

- ☐ My dog is strictly on a leash
- ☐ My dog exclusively runs free
- ☐ Both

3.3.0 [continued]

3.8 Does your dog go swimming in summer?

- ☐ No, he is very afraid of water
- ☐ Yes, but rarely (less than once a week)
- ☐ Yes, often

3.9 If yes, how does the dog enter the water?

- ☐ Only with the paws
- ☐ Dog swims completely

3.10 What is the purpose of use of your dog? (several answer options)

- ☐ Family dog
- ☐ Breeding dog
- ☐ Hunting dog
- ☐ Sports dog
- ☐ Guardian dog
- ☐ Working dog (e.g. rescue dog, police dog)
- ☐ Assistance dog (e.g. guide dog, diabetic warning dog)

3.11 Please mark with a cross whether your dog has visited one or more of the following possibilities in the last 12 months.

- ☐ Boarding kennel or dog sitter
- ☐ Dog parade or dog show
- ☐ Dog school
- ☐ Dog sports club
- ☐ None of the above mentioned

3.12 Please mark with a cross whether your dog has visited one or more of the following possibilities in the last 24 months.

- ☐ Boarding kennel or dog sitter
- ☐ Dog parade or dog show
- ☐ Dog school
- ☐ Dog sports
- ☐ None of the above mentioned

3.13 Please mark with a cross whether your dog has visited one or more of the following possibilities in the last 36 months.

- ☐ Boarding kennel or dog sitter
- ☐ Dog parade or dog show
- ☐ Dog school
- ☐ Dog sports
- ☐ None of the above mentioned

3.14 Has your dog been abroad in the last few months?

- ☐ Yes, in the previous 12 months
- ☐ Yes, in the previous 24 months
- ☐ Yes, in the previous 36 months
- ☐ Yes, but more than 36 months ago
- ☐ No, he has not been abroad in the past 36 months

3.15 Is your dog currently receiving medication to treat a disease?

- ☐ Yes
- ☐ No
- ☐ Unknown

3.16 If yes, which medication is your dog given for which disease?

4.0 Are you planning to take your dog with you to the following options in the next 36 months?

	Yes	No	Perhaps
4.1 Boarding kennel or dog sitter	☐	☐	☐
4.2 Dog parade or dog show	☐	☐	☐
4.3 Dog school	☐	☐	☐
4.4 Dog sports club	☐	☐	☐

5.0

5.1 Do you plan to take your dog abroad in the next 36 months?

☐ Yes, within the EU ☐ Yes, outside of the EU ☐ No

☐ Not sure

5.2 If so, to which country do you travel?

[ ]

5.3 Has your dog ever received a vaccination? ☐ Yes ☐ No

6.0 Please give an assessment of the importance of the vaccinations listed below in your opinion:

	Very important	Important	Less import	Unimportant	Unknown to me
6.1 Leptospirosis	☐	☐	☐	☐	☐
6.2 Kennel cough complex	☐	☐	☐	☐	☐
6.3 Leishmaniasis	☐	☐	☐	☐	☐
6.4 Hepatitis contagiosa canis	☐	☐	☐	☐	☐
6.5 Canine distemper	☐	☐	☐	☐	☐
6.6 Parvovir	☐	☐	☐	☐	☐
6.7 Canines herpes virus	☐	☐	☐	☐	☐
6.8 Rabies	☐	☐	☐	☐	☐
6.9 Skin mycosis	☐	☐	☐	☐	☐
6.10 Borreliosis	☐	☐	☐	☐	☐

7.0

7.1 When, if at all, did your dog receive his last leptospirosis vaccination or booster (refresher)?

☐ Within the past year ☐ More than 1 year to 3 years ago ☐ Over 3 years ago

☐ Never ☐ I do not know

7.0 [continued]

7.2 When, if at all, did your dog receive his last rabies vaccination or booster (refresher)?

- ☐ Within the past year
- ☐ More than 1 year to 3 years ago
- ☐ Over 3 years ago
- ☐ Never
- ☐ I do not know

7.3 When, if at all, did your dog receive his last distemper, hepatitis contagiosa canis, parvovirosis vaccination or booster (refresher)?

- ☐ Within the past year
- ☐ More than 1 year to 3 years ago
- ☐ Over 3 years ago
- ☐ Never
- ☐ I do not know

7.4 At what intervals is your dog vaccinated against leptospirosis?

- ☐ Each year
- ☐ Every two years
- ☐ Every 3 years
- ☐ Less often than every 3 years
- ☐ Never
- ☐ I do not know

7.5 At what intervals is your dog vaccinated against rabies?

- ☐ Each year
- ☐ Every two years
- ☐ Every 3 years
- ☐ Less often than every 3 years
- ☐ Never
- ☐ I do not know

7.6 At what intervals is your dog vaccinated against distemper, hepatitis contagiosa canis, parvovirosis?

- ☐ Each year
- ☐ Every two years
- ☐ Every 3 years
- ☐ Less often than every 3 years
- ☐ Never
- ☐ I do not know

8.0 Please tick in each case whether you found the following options helpful when informing about vaccinations:

	Very helpful	Helpful	Not helpful	Source not used
8.1 Veterinarian	☐	☐	☐	☐
8.2 Homeopathic practitioner	☐	☐	☐	☐
8.3 Internet	☐	☐	☐	☐
8.4 Books, magazines	☐	☐	☐	☐
8.5 Friends, relatives, colleagues	☐	☐	☐	☐
8.6 Breeder	☐	☐	☐	☐
8.7 Dog school	☐	☐	☐	☐
8.8 Pet shop	☐	☐	☐	☐

8.9 Others: ____________________

9.0

9.1 How well do you feel informed about dog vaccinations?

- ☐ Excellent, I think I am informed about everything important
- ☐ Average, I think I know a lot but it could be more
- ☐ Somewhat poor, I don't know much about vaccinations
- ☐ Poor, I don't know anything about vaccinations

9.2 Do you wish to receive more education and information about vaccinations from your veterinarian?

- ☐ Yes
- ☐ No

9.3 Which revaccination intervals has your veterinarian recommended for leptospirosis?

- ☐ Each year
- ☐ Every two years
- ☐ Every 3 years
- ☐ Less than every 3 years
- ☐ Only if necessary, after antibody measurement
- ☐ Never
- ☐ I do not know

9.3 Which revaccination intervals has your veterinarian recommended for rabies?

- ☐ Each year
- ☐ Every two years
- ☐ Every 3 years
- ☐ Less than every 3 years
- ☐ Only if necessary, after antibody measurement
- ☐ Never
- ☐ I do not know

9.3 Which revaccination intervals has your veterinarian recommended for distemper, hepatitis contagiosa canis, parvovirosis?

- ☐ Each year
- ☐ Every two years
- ☐ Every 3 years
- ☐ Less than every 3 years
- ☐ Only if necessary, after antibody measurement
- ☐ Never
- ☐ I do not know

10.0 Please evaluate how important the following points are for you when deciding whether to vaccinate your dog or not (*1= unimportant and 5= very important)*

	1	2	3	4	5
10.1 Expenses	☐	☐	☐	☐	☐
10.2 Side effects	☐	☐	☐	☐	☐
10.3 A stressful experience for the dog	☐	☐	☐	☐	☐
10.4 The vaccination is required for a vaccination certificate (e.g., for dog pesion, dog show, etc.)	☐	☐	☐	☐	☐
10.5 The vaccination is required for a trip abroad	☐	☐	☐	☐	☐
10.6 Veterinary advice	☐	☐	☐	☐	☐
10.7 The likelihood that your dog may develop this disease	☐	☐	☐	☐	☐
10.8 The effectiveness of vaccination	☐	☐	☐	☐	☐
10.9 The severity of the diseases against which the vaccination protects	☐	☐	☐	☐	☐
10.10 Time and inconvenience	☐	☐	☐	☐	☐
10.11 The age of the dog	☐	☐	☐	☐	☐

11.0

11.1 Which of the following points would prevent you from making a routine appointment with your veterinarian for a vaccination? (multiple answers possible)

- ☐ Opening hours
- ☐ Time expenses
- ☐ Stress during visit or transport
- ☐ Expenses
- ☐ Potential side effects
- ☐ None of the above
- ☐ Not applicable as the dog is not vaccinated
- ☐ Others

11.2 Please state your other reasons:

12.0 Section B

Section B

In this section you should consider all dogs that you own or have owned.

13.0 Please state whether one of your dogs has or has had the following infectious diseases: distemper, parvovirosis, leptospirosis, rabies, hepatitis contagiosa canis

13.1 I have/had an unvaccinated dog with one of these diseases ☐ Yes ☐ Not sure ☐ No

13.2 I have/had a vaccinated dog with one of these diseases ☐ Yes ☐ Not sure ☐ No

13.3 If yes, please state which of these diseases your unvaccinated dog is or was suffering from:

13.4 If yes, please state which of these diseases your vaccinated dog is or was suffering from:

14.0

14.1 Has a vaccination ever caused side effects in your dog? ☐ Yes ☐ No

14.2 What kind of side effects did your dog have?

- ☐ Lethargy
- ☐ Fever
- ☐ Lameness
- ☐ Others
- ☐ Inappetence
- ☐ Vomiting
- ☐ Anaphylactic reaction (within 24 hours)
- ☐ Injection site reaction
- ☐ Diarrhoea
- ☐ Immune mediated disease

14.3 Which immune-mediated disease was caused by the vaccination?

14.0 [continued]

14.4 What other side effects have been observed in your dog?

14.5 These side effects were:

☐ Insignificant and rare
☐ Insignificant and often
☐ Serious and rare
☐ Serious and often

14.6 Did these side effects prevent you from further vaccinations?

☐ Yes
☐ No

15.0 Section D

Section C.

The last section contains questions about you and your household. This information helps us to compare opinions about "dogs' vaccinations" with a cross-section of German households. Your data will not be used for other purposes. If there are questions you do not want to answer, leave the answer options blank and proceed to the next question.

15.1 What is your gender?
☐ Female ☐ Male

15.2 In which federal state do you live?

☐ Baden-Wuerttemberg ☐ Bavaria ☐ Berlin
☐ Brandenburg ☐ Bremen ☐ Hamburg
☐ Hesse ☐ Mecklenburg-Western Pomerania ☐ Lower Saxony
☐ North Rhine-Westphalia ☐ Rhineland-Palatinate ☐ Saarland
☐ Saxon ☐ Saxony-Anhalt ☐ Schleswig-Holstein
☐ Thuringia

15.3 Your residential area is located:
☐ In rural areas (under 50,000 inhabitants) ☐ In the catchment area of a city (50,000 to 500,000 inhabitants) ☐ In the catchment area of a large city (from 500,000 inhabitants)

15.4 What is the highest educational level in your household?
☐ Lower secondary school certificate ☐ General secondary school certificate ☐ Higher education entrace qualification
☐ University degree ☐ No answer

15.5 Your annual income is:
☐ Less than 10,000 Euros ☐ Between 10,000 and 25,000 Euros ☐ Between 25,000 and 50,000 Euros
☐ Between 50,000 and 75,000 Euros ☐ Between 75,000 and 100,000 Euros ☐ Over 100,000 Euros

15.6 Do you have children?
☐ Yes ☐ No

15.7 How many children under 18 years live in your household?
☐ None ☐ 1 ☐ 2
☐ 3 ☐ 4 or more

15.8 Was your youngest child vaccinated within the first year of life?
☐ Yes ☐ No

15.0 Section C [continued]

15.9 How old are you?

15.10 What is your attitude towards vaccinations?

- ☐ I think vaccinations as disease prevention are very important or even indispensable
- ☐ I think vaccinations are useful, but I weigh up the pros and cons thoroughly
- ☐ I'm rather skeptical about vaccinations
- ☐ I am basically against all kind of vaccinations

15.11 I am generally against vaccinations for the following reasons (multiple answers possible):

- ☐ Vaccinations are needless and unnecessary
- ☐ Vaccinations are harmful to health; they weaken the immune system
- ☐ Vaccinations can trigger other illnesses
- ☐ Vaccinations are only for the benefit of doctors and the pharmaceutical industry
- ☐ Other reasons

15.12 Please state your other reasons:

15.13 Have you yourself been vaccinated against tetanus for the last 10 years? ☐ Yes ☐ No ☐ Unknown

15.13 Have you yourself been vaccinated against diphtheria for the last 10 years? ☐ Yes ☐ No ☐ Unknown

15.13 Have you yourself been vaccinated against pertussis for the last 10 years? ☐ Yes ☐ No ☐ Unknown

15.13 Have you yourself been vaccinated against influenza for the last 10 years? ☐ Yes ☐ No ☐ Unknown

16.0 Section D- Vaccination advice

Vaccination advice: VOLUNTARY and not mandatory

If you would like to receive a free vaccination advice from the Medical Clinic for Small Animals of the LMU, please send us photos or scans of the pages of your dog’s vaccination card showing a) all recent and previous vaccinations, and b) the dogs' data page (important is the date of birth). Important is to use the vaccination card of the dog on which you were referring to in the survey. Please email all to: **Simone.Eschle@campus.lmu.de** and please enter your own email address in the following text field so we can get back to you with results of the consultation and specific vaccination advice:

16.1 To receive a free vaccination advice, please enter your email address here (not mandatory):

17.0 Section E

17.1 Do you have suggestions for improvement as well as requests or further ideas?

Thank you for your cooperation!
