# Supplementary material for: Canine vaccination in Germany: A survey of owner attitudes and compliance
Source: PLoS One. 2020 Aug 27;15(8):e0238371. doi: 10.1371/journal.pone.0238371 (PMC7451643; doi:10.1371/journal.pone.0238371)
Supplement: S1 Table — (DOCX) [file pone.0238371.s003.docx]

**S1 Table. Demographic details of the participating dog owners (n=3,881).**

| **Demographic detail** | | **Frequency of responses** | **Percentage of responses** |
| --- | --- | --- | --- |
| Gender | Female | 3,606/3,847 | 93.7 |
|  | Male | 241/3,847 | 6.3 |
| Federal State of residence | Baden-Wuerttemberg | 459/3,881 | 11.8 |
|  | Bavaria | 742/3,881 | 19.1 |
|  | Berlin | 235/3,881 | 6.1 |
|  | Brandenburg | 120/3,881 | 3.1 |
|  | Bremen | 13/3,881 | 0.3 |
|  | Hamburg | 65/3,881 | 1.7 |
|  | Hesse | 263/3,881 | 6.8 |
|  | Mecklenburg-Western Pomerania | 45/3,881 | 1.2 |
|  | Lower Saxony | 373/3,881 | 9.6 |
|  | North Rhine-Westphalia | 915/3,881 | 23.6 |
|  | Rhineland-Palatinate | 190/3,881 | 4.9 |
|  | Saarland | 44/3,881 | 1.1 |
|  | Saxon | 106/3,881 | 2.7 |
|  | Saxony-Anhalt | 74/3,881 | 1.9 |
|  | Schleswig-Holstein | 176/ 3,881 | 4.5 |
|  | Thuringia | 61/3,881 | 1.6 |
| Living area | Rural <50,000 inhabitants | 2,095/3,866 | 54.2 |
|  | City 50,000 to 500,000 inhabitants | 1,007/3,866 | 26.0 |
|  | Large city >500,000 inhabitants | 764/3,866 | 19.8 |
| Level of education in the household of the respondent | Lower secondary school certificate | 131/3,860 | 3.4 |
|  | General secondary school certificate | 967/3,860 | 25.1 |
|  | Higher education entrance qualification | 1,204/3,860 | 31.2 |
|  | University degree | 1,552/3,860 | 40.2 |
|  | No answer | 6/3,860 | 0.2 |
| Annual income | <10,000 € | 298/3,591 | 8.3 |
|  | 10,000 to 25,000 € | 887/3,591 | 24.7 |
|  | 25,000 to 50,000 € | 1,329/3,591 | 37.0 |
|  | 50,000 to 75,000 € | 610/3,591 | 17.0 |
|  | 75,000 to 100,000 € | 298/3,591 | 8.3 |
|  | >100,000 € | 169/3,591 | 4.7 |
| Children | Yes | 1,630/3,849 | 42.3 |
|  | No | 2,219/3,849 | 57.7 |

€ = Euro

The table contains all demographic information provided by the owners in the survey.
