# Supplementary material for: Canine vaccination in Germany: A survey of owner attitudes and compliance
Source: PLoS One. 2020 Aug 27;15(8):e0238371. doi: 10.1371/journal.pone.0238371 (PMC7451643; doi:10.1371/journal.pone.0238371)
Supplement: S2 Table — (DOCX) [file pone.0238371.s004.docx]

| **Question** | **Response option** | | **Frequency of responses** | **Percentage of responses** |
| --- | --- | --- | --- | --- |
| Age of the dog at acquisition | <8 weeks | | 199/3,873 | 5.1 |
|  | 8 to 16 weeks | | 2,295/3,873 | 59.3 |
|  | 16 weeks to 15 months | | 685/3,873 | 17.7 |
|  | 15 months to 5 years | | 505/3,873 | 13.0 |
|  | 5 to 10 years | | 154/3,873 | 4.0 |
|  | ≥10 years | | 20/3,873 | 0.5 |
|  | Unknown | | 15/3,873 | 0.4 |
| Origin of the dog | Animal shelter or charity abroad | | 708/3,875 | 18.3 |
|  | Animal shelter or charity in Germany | | 333/3,875 | 8.6 |
|  | Recognized breeder or breeding association | | 1,533/3,875 | 39.6 |
|  | Private or hobby breeder | | 608/3,875 | 15.7 |
|  | Newspaper advertisement | | 59/3,875 | 1.5 |
|  | Internet | | 245/3,875 | 6.3 |
|  | Friends, relatives, neighbours | | 204/3,875 | 5.3 |
|  | Other | | 185/3,875 | 4.8 |
| Purebred dog | Yes | | 2,432/3,885 | 62.7 |
|  | No | | 1,351/3,885 | 34.8 |
|  | Unknown | | 98/3,885 | 2.5 |
| Outdoor access | Strictly on a leash | | 398/3,877 | 10.3 |
|  | Runs exclusively free | | 296/3,877 | 7.6 |
|  | Both | | 3,183/3,877 | 82.1 |
| Kind of water contact | Only with the paws | | 1,042/3,840 | 27.1 |
|  | Full body | | 1,889/3,840 | 49.2 |
|  | Never | | 909/3,840 | 23.7 |
| Type of dog | Breeding dog | Yes | 362/3,881 | 9.3 |
|  |  | No | 3,519/3,881 | 90.7 |
|  | Hunting dog | Yes | 89/3,881 | 2.3 |
|  |  | No | 3,792/3,881 | 97.7 |
|  | Sports dog | Yes | 956/ 3,881 | 24.6 |
|  |  | No | 2,925/3,881 | 75.4 |
|  | Guardian dog | Yes | 212/3,881 | 5.5 |
|  |  | No | 3,669/3,881 | 94.5 |
|  | Assistance dog | Yes | 96/3,881 | 2.5 |
|  |  | No | 3,785/3,881 | 97.5 |
| Currently on medication | Yes | | 695/3,874 | 17.9 |
|  | No | | 3,179/3,874 | 82.1 |

**S2 Table. Characteristics of dogs owned by respondents participating in the web-based questionnaire (factors eliminated by the model) (n=3,881).**

The factors were included in the statistical analysis but they were not selected and therefore eliminated.
