# Supplementary material for: Canine vaccination in Germany: A survey of owner attitudes and compliance
Source: PLoS One. 2020 Aug 27;15(8):e0238371. doi: 10.1371/journal.pone.0238371 (PMC7451643; doi:10.1371/journal.pone.0238371)
Supplement: S3 Table — (DOCX) [file pone.0238371.s005.docx]

**S3 Table. Past and future outings of dogs owned by respondents (factors eliminated by the model) (n=3,881).**

| **Question** | **Response option** | | **Frequency of responses** | **Percentage of responses** |
| --- | --- | --- | --- | --- |
| Outings in the previous 12 months | Boarding kennel or dog sitter | Yes | 558/3,881 | 14.4 |
|  |  | No | 3,303/3,881 | 85.1 |
|  |  | Unknown | 20/3,881 | 0.5 |
|  | Dog show | Yes | 388/3,881 | 10.0 |
|  |  | No | 3,473/3,881 | 89.5 |
|  |  | Unknown | 20/3,881 | 0.5 |
|  | Dog training | Yes | 1,276/3,881 | 32.9 |
|  |  | No | 2,585/3,881 | 66.6 |
|  |  | Unknown | 20/3,881 | 0.5 |
|  | Dog sports club | Yes | 861/3,881 | 22.2 |
|  |  | No | 3,000/3,881 | 77.3 |
|  |  | Unknown | 20/3,881 | 0.5 |
| Outings in the previous 24 months | Dog show | Yes | 455/3,881 | 11.7 |
|  |  | No | 3,333/3,881 | 85.9 |
|  |  | Unknown | 93/3,881 | 2.4 |
|  | Dog training | Yes | 1,379/3,881 | 35.5 |
|  |  | No | 2,409/3,881 | 62.1 |
|  |  | Unknown | 93/3,881 | 2.4 |
|  | Dog sports club | Yes | 894/3,881 | 23.0 |
|  |  | No | 2,894/3,881 | 74.6 |
|  |  | Unknown | 93/3,881 | 2.4 |
| Outings in the previous 36 months | Boarding kennel or dog sitter | Yes | 562/3,881 | 14.5 |
|  |  | No | 3,145/3,881 | 81.0 |
|  |  | Unknown | 174/3,881 | 4.5 |
|  | Dog show | Yes | 467/3,881 | 12.0 |
|  |  | No | 3,240/3,881 | 83.5 |
|  |  | Unknown | 174/3,881 | 4.5 |
|  | Dog training | Yes | 1,354/3,881 | 34.9 |
|  |  | No | 2,353/3,881 | 60.6 |
|  |  | Unknown | 174/3,881 | 4.5 |
|  | Dog sports club | Yes | 854/3,881 | 22.0 |
|  |  | No | 2,853/3,881 | 73.5 |
|  |  | Unknown | 174/3,881 | 4.5 |
| Outing to dog sports club (in the next 36 months) | Yes | | 995/3,729 | 26.7 |
|  | No | | 2,215/3,729 | 59.4 |
|  | Unknown | | 519/3,729 | 13.9 |
| Stay abroad for the next 36 months | Yes, inside the EU | | 1,851/3,852 | 48.1 |
|  | Yes, outside the EU | | 93/3,852 | 2.4 |
|  | No | | 1,372/3,852 | 35.6 |
|  | Unknown | | 536/3,852 | 13.9 |
| Destination of the planned trip | Northern country | Yes | 444/3,881 | 11.4 |
|  |  | No | 2,065/388 | 53.2 |
|  |  | No trip abroad planned | 1,372/3,881 | 35.4 |
|  | Eastern country | Yes | 699/3,881 | 18.0 |
|  |  | No | 1,810/3,881 | 46.6 |
|  |  | No trip abroad planned | 1,372/3,881 | 35.4 |
|  | Southern country | Yes | 1,177/3,881 | 30.3 |
|  |  | No | 1,332/3,881 | 34.3 |
|  |  | No trip abroad planned | 1,372/3,881 | 35.4 |
|  | Western country | Yes | 895/3,881 | 23.1 |
|  |  | No | 1,614/3,881 | 41.6 |
|  |  | No trip abroad planned | 1,372/3,881 | 35.4 |

EU = European Union

The factors were included in the statistical analysis but they were not selected and therefore eliminated.
