# Supplementary material for: Canine vaccination in Germany: A survey of owner attitudes and compliance
Source: PLoS One. 2020 Aug 27;15(8):e0238371. doi: 10.1371/journal.pone.0238371 (PMC7451643; doi:10.1371/journal.pone.0238371)
Supplement: S4 Table — (DOCX) [file pone.0238371.s006.docx]

**S4 Table. Source of information regarding canine vaccination and attitude toward vaccination of the participating owners (factors eliminated by the model) (n=3,881).**

| **Question** | **Response option** | | **Frequency of responses** | **Percentage of responses** |
| --- | --- | --- | --- | --- |
| Source of vaccination information | Internet | Very helpful | 564/3,787 | 14.9 |
|  |  | Helpful | 1,538/3,787 | 40.6 |
|  |  | Not helpful | 582/3,787 | 15.4 |
|  |  | Source not used | 1,103/3,787 | 29.1 |
|  | Friends, relatives, colleagues | Very helpful | 201/3,730 | 5.4 |
|  |  | Helpful | 895/3,730 | 24.0 |
|  |  | Not helpful | 952/3,730 | 25.5 |
|  |  | Source not used | 1,682/3,730 | 45.1 |
|  | Breeder | Very helpful | 402/3,773 | 10.7 |
|  |  | Helpful | 692/3,773 | 18.3 |
|  |  | Not helpful | 482/3,773 | 12.8 |
|  |  | Source not used | 2,197/3,773 | 58.2 |
|  | Dog trainer | Very helpful | 164/3,739 | 4.4 |
|  |  | Helpful | 464/3,739 | 12.4 |
|  |  | Not helpful | 574/3,739 | 15.4 |
|  |  | Source not used | 2,537/3,739 | 67.9 |
|  | Pet shop | Very helpful | 13/3,695 | 0.4 |
|  |  | Helpful | 41/3,695 | 1.1 |
|  |  | Not helpful | 590/3,695 | 16.0 |
|  |  | Source not used | 3,051/3,695 | 82.6 |
| Need for more information from the veterinarian | Yes | | 2,204/3,851 | 57.2 |
|  | No | | 1,647/3,851 | 42.8 |
| Factors influencing owner’s decision to have dog vaccinated | Expenses | Unimportant | 2,805/3,861 | 72.6 |
|  |  | Somewhat unimportant | 449/3,861 | 11.6 |
|  |  | Somewhat important | 333/3,861 | 8.6 |
|  |  | Important | 132/3,861 | 3.4 |
|  |  | Very important | 142/3,861 | 3.7 |
|  | Stressful experience for the dog | Unimportant | 826/3,837 | 21.5 |
|  |  | Somewhat unimportant | 595/3,837 | 15.5 |
|  |  | Somewhat important | 1,057/3,837 | 27.5 |
|  |  | Important | 479/3,837 | 12.5 |
|  |  | Very important | 880/3,837 | 22.9 |
|  | Required for vaccination certificate | Unimportant | 1,357/3,845 | 35.3 |
|  |  | Somewhat unimportant | 275/3,845 | 7.2 |
|  |  | Somewhat important | 604/3,845 | 15.7 |
|  |  | Important | 486/3,845 | 12.6 |
|  |  | Very important | 1,123/3,845 | 29.2 |
|  | Required for trip abroad | Unimportant | 853/3,852 | 22.1 |
|  |  | Somewhat unimportant | 142/3,852 | 3.7 |
|  |  | Somewhat important | 438/3,852 | 11.4 |
|  |  | Important | 492/3,852 | 12.8 |
|  |  | Very important | 1,927/3,852 | 50.0 |
|  | Risk of infection | Unimportant | 375/3,851 | 9.7 |
|  |  | Somewhat unimportant | 221/3,851 | 5.7 |
|  |  | Somewhat important | 661/3,851 | 17.2 |
|  |  | Important | 711/3,851 | 18.5 |
|  |  | Very important | 1,883/3,851 | 48.9 |
|  | Effectiveness of the vaccine | Unimportant | 314/3,854 | 8.1 |
|  |  | Somewhat unimportant | 116/3,854 | 3.0 |
|  |  | Somewhat important | 450/3,854 | 11.7 |
|  |  | Important | 707/3,854 | 18.3 |
|  |  | Very important | 2,267/3,854 | 58.8 |
|  | Time expenditures and inconvenience | Unimportant | 2,611/3,846 | 67.9 |
|  |  | Somewhat unimportant | 433/3,846 | 11.3 |
|  |  | Somewhat important | 388/3,846 | 10.1 |
|  |  | Important | 133/3,846 | 3.5 |
|  |  | Very important | 281/3,846 | 7.3 |
| Potential deterrents from making a veterinary appointment | Opening hours | Yes | 259/3,881 | 6.7 |
|  |  | No | 3,622/3,881 | 93.3 |
|  | Time expenditure | Yes | 102/3,881 | 2.6 |
|  |  | No | 3,779/3,881 | 97.4 |
|  | Stress during visit or transport | Yes | 472/3,881 | 12.2 |
|  |  | No | 3,409/3,881 | 87.8 |
|  | Expense | Yes | 249/3,881 | 6.4 |
|  |  | No | 3,632/3,881 | 93.6 |
|  | Potential VAAEs | Yes | 2,093/3,881 | 53.9 |
|  |  | No | 1,788/3,881 | 46.1 |
| Infected dog (CDV, CPV, RV, *Leptospira*) without vaccination | Yes | | 54/3,800 | 1.4 |
|  | Uncertain | | 20/3,800 | 0.5 |
|  | No | | 3,726/3,800 | 98.1 |
| Infected dog (CDV, CPV, RV, *Leptospira*) despite vaccination | Yes | | 159/3,854 | 4.1 |
|  | Uncertain | | 25/3,854 | 0.6 |
|  | No | | 3,670/3,854 | 95.2 |
| VAAEs in the past | Yes | | 1,613/3,867 | 41.7 |
|  | No | | 2,254/3,867 | 58.3 |

VAAEs = vaccine-associated adverse effects; CPV = canine parvovirus; CDV = canine distemper virus; RV = rabies virus

The factors were included in the statistical analysis but they were not selected and therefore eliminated.
