# Supplementary material for: Canine vaccination in Germany: A survey of owner attitudes and compliance
Source: PLoS One. 2020 Aug 27;15(8):e0238371. doi: 10.1371/journal.pone.0238371 (PMC7451643; doi:10.1371/journal.pone.0238371)
Supplement: S5 Table — (DOCX) [file pone.0238371.s007.docx]

**S5 Table. Sociodemographic details of the participating owners (factors eliminated by the model) (n=3,881).**

| **Question** | **Response option** | | **Frequency of responses** | **Percentage of responses** |
| --- | --- | --- | --- | --- |
| Federal State of residence | Baden-Wuerttemberg | | 459/3,881 | 11.8 |
|  | Bavaria | | 742/3,881 | 19.1 |
|  | Berlin | | 235/3,881 | 6.1 |
|  | Brandenburg | | 120/3,881 | 3.1 |
|  | Bremen | | 13/3,881 | 0.3 |
|  | Hamburg | | 65/3,881 | 1.7 |
|  | Hesse | | 263/3,881 | 6.8 |
|  | Mecklenburg-Western Pomerania | | 45/3,881 | 1.2 |
|  | Lower Saxony | | 373/3,881 | 9.6 |
|  | North Rhine-Westphalia | | 915/3,881 | 23.6 |
|  | Rhineland-Palatinate | | 190/3,881 | 4.9 |
|  | Saarland | | 44/3,881 | 1.1 |
|  | Saxon | | 106/3,881 | 2.7 |
|  | Saxony-Anhalt | | 74/3,881 | 1.9 |
|  | Schleswig-Holstein | | 176/ 3,881 | 4.5 |
|  | Thuringia | | 61/3,881 | 1.6 |
| Living area | Rural <50,000 inhabitants | | 2,095/3,866 | 54.2 |
|  | City 50,000 to 500,000 inhabitants | | 1,007/3,866 | 26.0 |
|  | Large city >500,000 inhabitants | | 764/3,866 | 19.8 |
| Level of education in the household | Lower secondary school certificate | | 131/3,860 | 3.4 |
|  | General secondary school certificate | | 967/3,860 | 25.1 |
|  | Higher education entrance qualification | | 1,204/3,860 | 31.2 |
|  | University degree | | 1,552/3,860 | 40.2 |
|  | No answer | | 6/3,860 | 0.2 |
| Annual income | <10,000 € | | 298/3,591 | 8.3 |
|  | 10,000 to 25,000 € | | 887/3,591 | 24.7 |
|  | 25,000 to 50,000 € | | 1,329/3,591 | 37.0 |
|  | 50,000 to 75,000 € | | 610/3,591 | 17.0 |
|  | 75,000 to 100,000 € | | 298/3,591 | 8.3 |
|  | >100,000 € | | 169/3,591 | 4.7 |
| Children | Yes | | 1,630/3,849 | 42.3 |
|  | No | | 2,219/3,849 | 57.7 |
| Youngest child vaccinated within the first year of life | Yes | | 1,461/3,824 | 38.2 |
|  | No | | 144/3,824 | 3.8 |
|  | No children | | 2,219/3,824 | 58.0 |
| Respondent was vaccinated in the previous 10 years | Pertussis | Yes | 1,055/3,864 | 27.3 |
|  |  | No | 2,406/3,864 | 62.3 |
|  |  | Unknown | 403/3,864 | 10.4 |
|  | Influenza | Yes | 653/3,877 | 16.8 |
|  |  | No | 3,134/3,877 | 80.8 |
|  |  | Unknown | 90/3,877 | 2.3 |

€ = Euro

The factors were included in the statistical analysis but they were not selected and therefore eliminated.
